# Monoaminergic Orchestration of Motor Programs in a Complex *C. elegans* Behavior

**DOI:** 10.1371/journal.pbio.1001529

**Published:** 2013-04-02

**Authors:** Jamie L. Donnelly, Christopher M. Clark, Andrew M. Leifer, Jennifer K. Pirri, Marian Haburcak, Michael M. Francis, Aravinthan D. T. Samuel, Mark J. Alkema

**Affiliations:** 1 Department of Neurobiology, University of Massachusetts Medical School, Worcester, Massachusetts, United States of America; 2 Department of Physics & Center for Brain Science, Harvard University, Cambridge, Massachusetts, United States of America; University of Utah, United States of America

## Abstract

Monoamines provide chemical codes of behavioral states. However, the neural mechanisms of monoaminergic orchestration of behavior are poorly understood. Touch elicits an escape response in *Caenorhabditis elegans* where the animal moves backward and turns to change its direction of locomotion. We show that the tyramine receptor SER-2 acts through a Gα_o_ pathway to inhibit neurotransmitter release from GABAergic motor neurons that synapse onto ventral body wall muscles. Extrasynaptic activation of SER-2 facilitates ventral body wall muscle contraction, contributing to the tight ventral turn that allows the animal to navigate away from a threatening stimulus. Tyramine temporally coordinates the different phases of the escape response through the synaptic activation of the fast-acting ionotropic receptor, LGC-55, and extrasynaptic activation of the slow-acting metabotropic receptor, SER-2. Our studies show, at the level of single cells, how a sensory input recruits the action of a monoamine to change neural circuit properties and orchestrate a compound motor sequence.

## Abstract 2

A single monoamine can orchestrate different phases of a compound motor sequence in *C. elegans* through the synaptic and extra-synaptic activation of distinct classes of receptors.

## Author Summary

How the nervous system controls complex behaviors has intrigued neurobiologists for decades. There are many examples where sequential motor patterns of specific behaviors have been described in great detail. However, the neural mechanisms that orchestrate a full behavioral sequence are poorly understood. Gentle touch to the head of the roundworm *C. elegans* elicits an escape response in which the animal quickly moves backward. The reversal is followed by a deep turn that allows the animal to change its direction of locomotion and move away from the threatening stimulus. We found that the neurotransmitter tyramine controls the initial reversal phase of the escape response through the activation of a fast-acting ion channel and the later turning phase through the activation of a slow-acting G-protein coupled receptor (GPCR). We show that this tyramine GPCR is expressed in neurons that make contacts with the ventral muscles of the animal. Activation of this receptor facilitates the contraction of ventral muscles and thus allows the animal to turn and resume locomotion in the opposite direction during its escape. Our studies show how a single neurotransmitter coordinates sequential phases of a complex behavior through the activation of distinct classes of receptors.

## Introduction

Complex behaviors require the temporal coordination of independent motor programs in which neurotransmitters and neuromodulators orchestrate the output of neural circuits. How the nervous system directs sequential activation and inhibition of assemblies of neurons, however, is largely unclear. Neurotransmitters can directly activate ligand-gated ion channels at synapses, inducing rapid changes in the electrical activity of postsynaptic cells. Neuromodulators generally act through G-protein coupled receptors (GPCR) that activate intracellular signaling cascades with slower but longer lasting effects. The release of neuromodulators can activate or refine basic motor patterns generated by fast-acting neurotransmitters in a neural network [1]–[3].

In mammals, monoamines such as serotonin, dopamine, and noradrenaline are associated with specific behavioral states. Adrenergic modulation provides one of the most striking examples of the coordination of behavior and physiology to reflect an internal state of stress. Adrenergic transmitters stimulate the amygdala and increase heart rate, muscle tone, oxygen supply to the brain, and the release of glucose from energy stores to prepare for a fight-or-flight response [4]. Noradrenaline and adrenaline are not used by invertebrates, but the structurally related monoamines octopamine and tyramine are often considered as the invertebrate counterparts of these adrenergic transmitters [5]. Octopamine and tyramine have been implicated in subordinate behavior in lobsters [6], the honey bee sting response [7], the energy metabolism of flight in locusts [8], aggression in crickets and fruit flies [9]–[12], and the escape response of *C. elegans* [13]. Pioneering studies in the locust [14], mollusks [15], and crustaceans [16] have supported an “orchestration hypothesis” [17] where monoamines control behavioral states through the recruitment of distinct neural circuits. However, the sensory input that triggers the release of monoamines and the molecular and neural coding of these behaviors remain poorly understood.

The *C. elegans* escape response consists of a behavioral sequence used by the animal to navigate away from a threatening stimulus. *C. elegans* moves on its side by propagating a sinusoidal wave of ventral-dorsal muscle contractions along the length of its body [18]. Locomotion is normally accompanied by exploratory head movements in which the tip of the nose moves rapidly from side to side. Gentle touch to the head elicits a backing response and the suppression of exploratory head movements [13],[19]. The backing response is usually followed by a deep ventral head bend, allowing the animal to make a sharp (omega) turn and change its direction of locomotion. The completion of the entire escape response takes approximately 10 s and requires sensory processing, decision-making, and sequential inhibition and activation of distinct motor programs. Therefore, the anterior touch response is a highly orchestrated motor sequence with complexity far beyond that of a simple reflex.

The neural wiring diagram in combination with genetic and laser ablation experiments has provided a framework for the neural circuit that controls the initial phase of the escape response [19]–[21]. *C. elegans* has a single pair of tyraminergic motor neurons that are essential in coordinating the backing response with suppression of head movements [13]. Synaptic activation of the tyramine-gated chloride channel, LGC-55, inhibits forward locomotion and induces the relaxation of neck muscle [22]. The coordination of these motor programs increases the animals' chances of escaping from predacious fungi that use constricting rings to catch nematodes, illustrating the vital importance of monoaminergic motor control [23],[24]. How this initial phase of the escape response is temporally linked to later stages in which the animal makes a sharp turn to navigate away from the stimulus is unknown. To elucidate how monoamines may orchestrate the activity of specific neural circuits in complex behaviors, we analyzed the role of tyramine in *C. elegans* locomotion during the escape response. We show that the extrasynaptic activation of a G-protein coupled tyramine receptor generates asymmetry in a locomotion program, thus allowing the animal to execute a deep ventral turn and navigate away from the stimulus.

## Results

### *ser-2* Mutants Are Resistant to Exogenous Tyramine

**Figure 1:**
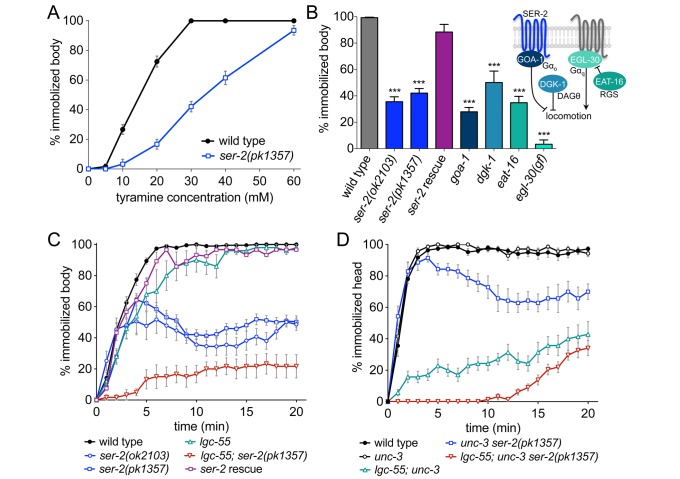
*ser-2* mutants are partially resistant to the paralytic effects of exogenous tyramine. (A) Tyramine induces immobilization in a dose-dependent manner. Shown is the percentage of animals immobilized after 10 min on agar plates supplemented with tyramine. Wild-type animals become fully immobilized at concentrations above 30 mM tyramine, while *ser-2* mutants continue sustained movement. Each data point represents the mean ± the standard error of the mean (SEM) for at least three trials, totaling a minimum of 30 animals. (B) G-protein signaling mutants are resistant to the paralytic of effects of exogenous tyramine. Shown is the percentage of animals that become immobilized after 10 min on 30 mM tyramine. Each bar represents the mean ± SEM for at least four trials totaling a minimum of 40 animals. (Inset) Schematic representation of the Gα_o_ and Gα_q_ signaling pathways that modulate locomotion in *C. elegans*. The genetic data suggest that SER-2 acts in the Gα_o_ pathway. The names of the human orthologs are shown. Rescue denotes the transgenic line *Pser-2*::SER-2; *ser-2(pk1357).* (C, D) Tyramine affects locomotion and head movements through different mechanisms. Shown is the percentage of animals with sustained body (C) or head (D) movements on 30 mM tyramine. *ser-2* mutants are partially resistant to the effects of tyramine on body movements, but not head movements. *lgc-55* mutants continue to move their heads through the duration of the assay. Each data point represents the mean percentage of animals that become immobilized by tyramine each minute for 20 min ± SEM for at least six trials, totaling a minimum of 60 animals. Head movements were analyzed in an *unc-3* mutant background. *unc-3* mutants make few body movements but display normal head movements, and are wild-type for the *ser-2 and lgc-55* loci. Statistical significance to wild-type: ***p<0.0001, two-tailed Student's *t* test. See also Figures S1 and S2.

*C. elegans* become immobilized on plates containing exogenous tyramine in a dose-dependent manner (Figure 1A and Figure S1) [22]. Three GPCRs have been shown to bind tyramine with high affinity: TYRA-2, TYRA-3, and SER-2 [25]–[28]. To determine whether the effects of tyramine are mediated by these GPCRs, we examined the locomotion of *ser-2(pk1357* and *ok2103)*, *tyra-2(tm1815)*, and *tyra-3(ok325)* deletion mutants on agar plates containing exogenous tyramine. Wild-type, *tyra-2*, and *tyra-3* mutant animals become immobilized on 30 mM tyramine within 5 min (Figure S2). However, *ser-2* mutant animals sustained movement on plates containing exogenous tyramine (Figure 1A–C and Figure S1). Sensitivity to exogenous tyramine is restored back to wild-type levels in *ser-2* mutants containing a *ser-2* genomic transgene (Figure 1B,C and Figure S2). This indicates that exogenous tyramine mediates its paralytic effects through the hyperactivation of endogenous tyramine signaling pathways that are at least in part dependent on SER-2.

We have previously shown that activation of the tyramine-gated chloride channel, LGC-55, inhibits head movements and forward locomotion [22]. Exogenous tyramine initially induced backward locomotion and the inhibition of head movements in both the wild-type *and ser-2* mutants. However, unlike the wild type, *ser-2* mutants recovered and resumed forward locomotion and head movements within minutes. Body movements of *lgc-55* mutants are inhibited, similar to the wild-type, but head movements are sustained on exogenous tyramine (Figure 1C,D). *lgc-55; ser-2* double mutants largely persisted both head and body movements on exogenous tyramine, however locomotion remained slightly uncoordinated. This indicates that while the activation of the ionotropic LGC-55 and metabotropic SER-2 receptor are required for paralysis, exogenous tyramine may affect locomotion through the activation of other receptors.

Head movements and body movements are controlled by distinct groups of muscles and motor neurons [13],[20]. In wild-type animals the inhibition of head movements occurred rapidly (half time to immobilization, t_i50 = _77±2 s) and was followed by the slower inhibition of body movement (t_i50 = _149±3 s). This suggests that signaling pathways with distinct kinetics contribute to tyramine's effects. The sustained body movements of *ser-2* mutants on exogenous tyramine make it difficult to dissect the effects of exogenous tyramine on head movements. Therefore, we analyzed the effect of tyramine on head movements in an *unc-3(e151)* mutant background. *unc-3* mutants display few body movements, but have normal head movements [29]. Head movements of *ser-2 unc-3* double mutants were inhibited on exogenous tyramine, like those in the wild-type and *unc-3* mutants (Figure 1D). Consistent with our previous observation [22], head movements were sustained in *lgc-55: unc-3* double mutants. The kinetics and distinct inhibition of head and body movements of *lgc-55* and *ser-2* mutants indicates that exogenous tyramine induces the fast immobilization of head movements mainly through the hyperactivation of the ionotropic tyramine receptor LGC-55 followed by the immobilization of body movements through hyperactivation of the metabotropic tyramine receptor SER-2.

### Gα_o_ Signaling Pathway Mutants Are Resistant to Exogenous Tyramine

To identify genes involved in tyramine signaling, we performed a genetic screen for mutants that are resistant to the immobilizing effects of exogenous tyramine on body movements. Four isolates from this screen, *zf47*, *zf97*, *zf98*, *and zf133*, sustained movement on plates containing 30 mM tyramine. Genetic mapping, complementation tests, and sequence analysis showed that *zf47* was an allele of *goa-1. goa-1* encodes the *C. elegans* ortholog of the neural G protein-alpha subunit of the Gα_o_ class. GOA-1/Gα_o_ is expressed throughout the nervous system and is thought to negatively regulate synaptic transmission through the inhibition of EGL-30, the *C. elegans* Gα_q_ ortholog [30]–[32]. We found that *zf97*, *zf98*, and *zf133* were alleles of *dgk-1*, which encodes the *C. elegans* ortholog of the vertebrate brain diacylglycerol kinase theta (DAGθ) [32],[33]. *dgk-1* mutants have a reduced ability to deplete DAG generated by the *egl-30/*Gα_q_ pathway [33],[34]. Previously characterized null mutants for *goa-1* and *dgk-1* were also resistant to the paralytic effect of tyramine on both head and body movements (Figure 1B). We found that *egl-30*/Gα_q_ gain-of-function mutants and mutants for *eat-16*, which encodes a Regulator of G-protein Signaling (RGS) protein that inhibits EGL-30 [35], are resistant to exogenous tyramine (Figure 1B). Unlike *goa-1* and *dgk-1* mutants, which exhibit hyperactive locomotion and egg-laying behavior, *ser-2* mutants did not have obvious behavioral defects [36]. Therefore, mutations that reduce GOA-1/Gα_o_ signaling or increase EGL-30/Gα_q_ signaling confer resistance to exogenous tyramine. The resistance of *goa-1* and *ser-2* mutants to exogenous tyramine suggests that SER-2 signals through GOA-1/Gα_o_ to modulate locomotion.

### SER-2 Acts in GABAergic Neurons

**Figure 2:**
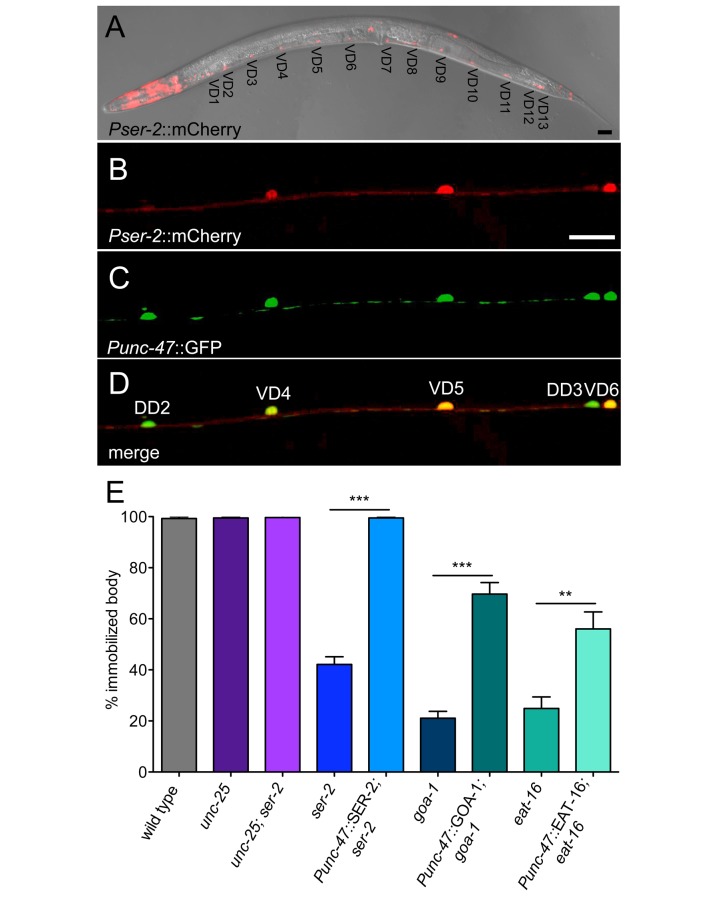
*ser-2* is expressed in a subset of GABAergic motor neurons. (A) A composite DIC image with fluorescent overlay showing that the *Pser*-2::mCherry transcriptional reporter is expressed in head muscles, head neurons, and neurons in the ventral nerve cord. (B–D) Transgenic animal showing coexpression of *Pser-2::*mCherry (B) and *Punc-47::*GFP, which labels all GABAergic motor neurons (C). *Pser-2::*mCherry is strongly expressed in the GABAergic VD neurons but not the DD neurons (D). Anterior is to the left. Scale bar is 20 µm. (E) Exogenous tyramine induces immobilization though the activation of SER-2 and Gα_o_ signaling pathway in the GABAergic neurons. Shown is the percentage of animals that become immobilized after 10 min on 30 mM tyramine. Loss-of-function of *unc-25* (glutamic acid decarboxylase) suppresses the tyramine resistance of *ser-2* mutant animals. *unc-25* (GABA deficient) mutants and *unc-25; ser-2(pk1357)* double mutants are not resistant to the paralytic effects of exogenous tyramine. Expression of SER-2 in all GABAergic neurons (*Punc-47*::SER-2) restores sensitivity of *ser-2* mutants to exogenous tyramine. Expression of GOA-1/Gα_o_ or EAT-16/RGS in all GABAergic neurons (*Punc-47*::GOA-1 or *Punc-47*::EAT-16) partially restores sensitivity to exogenous tyramine in the respective *goa-1* and *eat-16* mutants. Each bar represents the mean ± SEM for at least three trials, totaling a minimum of 30 animals.

Where does SER-2 act to confer resistance to exogenous tyramine? A *ser-2* genomic rescuing transgene restored sensitivity of *ser-2* mutants to exogenous tyramine, indicating that the transgene is expressed in the cells that confer sensitivity to exogenous tyramine (Figure 1B and Figure S2). The *ser-2* genomic locus is large and complex and encodes several splice variants [25],[36]. To analyze the *ser-2* expression pattern we expressed mCherry under the control of an 11.8 kb *ser-2* promoter sequence that included the four alternative first exons. We found that our *Pser-2*::mCherry reporter was expressed in head muscles, as well as several neurons in the head and ventral cord. The *ser-2* reporter was expressed in the first and second row of muscle cells, which are distinct from neck muscles (third and fourth row of muscle cells) that express the tyramine-gated chloride channel LGC-55 (Figure S3). *lgc-55* mutants fail to suppress head movements in response to touch, but head movements were suppressed normally in *ser-2* mutants, indicating that only LGC-55 is required for tyramine-induced head relaxation. The *ser-2* reporter was not expressed in interneurons that control locomotion, as expression did not overlap with *Pglr-1*::GFP and *Plgc-55*::GFP reporters that are expressed in the locomotion command neurons (unpublished data). *ser-2* reporter expression was also observed in 13 cells in the ventral cord (Figure 2A) that send commissures to the dorsal cord. The ventral cord is composed of excitatory cholinergic and inhibitory GABAergic motor neurons that innervate body wall muscles and control locomotion. Coexpression analysis with GFP reporters that specifically label cholinergic or GABAergic neurons showed that *Pser-2*::mCherry was highly expressed in a subset of GABAergic motor neurons (Figure 2B–D). The same set of GABAergic motor neurons were labeled in transgenic line (*Pser-2a*::GFP) that expressed a reporter for the SER-2A isoform (unpublished data) [36]. GABAergic ventral nerve cord neurons are subdivided into 13 VD motor neurons that synapse onto the ventral body wall muscles, and 6 DD motor neurons that synapse onto the dorsal body wall muscles. The cells that highly express the *ser-2* reporter do not co-label with a *Pflp-13*::GFP reporter, which is expressed in the DD motor neurons (unpublished data) [37]. This indicates SER-2 is specifically expressed in the GABAergic VD motor neurons.

Since GABAergic motor neurons are required for normal locomotion, we tested if SER-2 acts in these cells to mediate the inhibitory effects of tyramine on movement. No other promoters have been described that specifically drive expression in the GABAergic VD neurons. The complexity and size of the *ser-2* promoter did not allow us to define promoter elements that specifically drive expression in the VD neurons. We therefore expressed *ser-2* cDNA in all GABAergic neurons using the *unc-47* promoter (*Punc-47::*SER-2). *ser-2* expression in GABAergic neurons of *ser-2* mutants restored normal sensitivity to exogenous tyramine (Figure 2E).

To determine if GABA signaling affects tyramine sensitivity, we tested GABA-deficient mutants for sensitivity to exogenous tyramine. We found that *unc-25* mutants, which lack glutamate decarboxylase required for GABA synthesis [38], were slightly hypersensitive to the immobilizing effects of exogenous tyramine (Figure 2E, Figure S4). This suggests that reduced GABA signaling increases the sensitivity to exogenous tyramine, likely through the hyperactivation of other tyramine receptors, such as LGC-55 and TYRA-2. GABA deficiency suppressed the resistance phenotype of *ser-2* mutants, since *unc-25; ser-2* double mutants were nearly as sensitive to tyramine as *unc-25* single mutants (Figure 2E). These epistasis experiments indicate that SER-2 acts upstream or in parallel to GABAergic signaling and are consistent with the hypothesis that SER-2 acts in the VD neurons to inhibit GABA signaling and control locomotion.

We performed cell-specific rescue experiments to determine whether G-protein signaling components are required in the GABAergic neurons to mediate sensitivity to the exogenous tyramine. Expression of *goa-1* or *eat-16* in the GABAergic neurons (*Punc-47::*GOA-1/Gα_o_ and *Punc-47::*EAT-16/RGS) of *goa-1* and *eat-16* mutants, respectively, did not rescue the hyperactive locomotion phenotype. However, *goa-1* expression in GABAergic neurons of *goa-1* mutants largely restored sensitivity to exogenous tyramine (Figure 2E, Figure S4). Similarly, rescue expression of *eat-16* in GABAergic neurons partly restores sensitivity to exogenous tyramine. The activation of the Gα_q_ pathway in other cells may contribute to tyramine resistance phenotype in G-protein signaling mutants since the sensitivity to tyramine is not completely restored to wild-type levels. Nonetheless, the increased sensitivity of GABA-neuron-specific rescue of G-protein signaling mutants suggest that exogenous tyramine induces body immobilization through the activation of SER-2A and a Gα_o_ pathway in GABAergic neurons.

### SER-2 Inhibits Neurotransmitter Release

**Figure 3:**
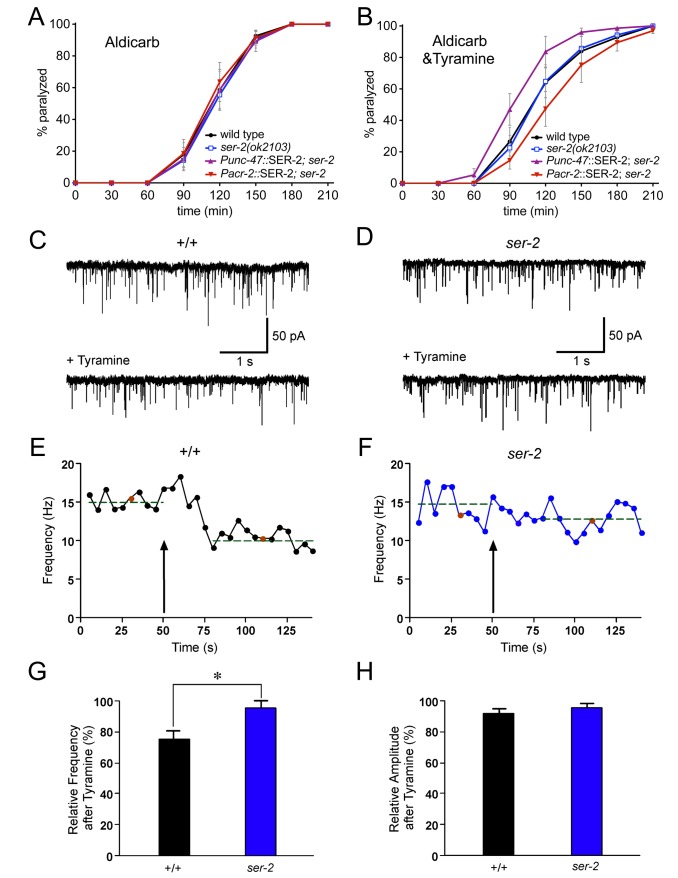
Tyramine-mediated reduction in GABA synaptic release requires SER-2. (A, B) SER-2 expression in cholinergic (*Pacr-2::*SER-2) or GABAergic (*Punc-47::*SER-2) motor neurons alters the rate of paralysis of *ser-2* mutants on aldicarb drug plates. All genotypes paralyze at a similar rate on drug plates containing only 0.5 mM aldicarb (A), yet drug plates containing both 30 mM tyramine and 0.5 mM aldicarb causes paralytic resistance (*Pacr-2::*SER-2) or hypersensitivity (*Punc-47::*SER-2) (B). Aldicarb experiments were conducted on nematode growth media (NGM) plates. 30 mM tyramine dissolved in NGM agar does not inhibit locomotion. SER-2 was expressed in *ser-2(pk1357)* mutant animals. Each data point represents the mean percentage of animals immobilized by aldicarb scored every 30 min ± SEM for at least four trials, totaling a minimum of 60 animals. (C, D) Representative endogenous inhibitory postsynaptic currents (IPSCs) recorded from ventral body wall muscles. +/+, *unc-29; acr-16* double mutants that lack excitatory neurotransmission at the NMJ are wild-type for the *ser-2* locus. *ser-2*, *unc-29; acr-16; ser-2(pk1357)* triple mutants. (E, F) Tyramine application decreased the rate of IPSCs in +/+ (*n* = 6), but not *ser-2* mutants (*n* = 5). Arrow depicts tyramine application time point. Each point represents the IPSC frequency calculated over a 5-s time window as indicated. The red points correspond to the displayed samples in (C) and (D), respectively. Dashed lines show average frequency before tyramine application and during the stabilized tyramine response period. (G) Average IPSC frequency after tyramine application plotted relative to IPSC frequency prior to tyramine exposure. Values were normalized to average frequency observed in control recordings in the absence of tyramine. (H) Average amplitude of IPSCs after tyramine application plotted relative to average amplitude prior to tyramine exposure. Error bars depict SEM. Statistical differences calculated from +/+: *p<0.05, two-tailed Student's *t* test.

To test the hypothesis that SER-2 modulates neurotransmitter release from ventral cord motor neurons, we analyzed mutants for their sensitivity to the acetylcholinesterase inhibitor aldicarb. Aldicarb increases acetylcholine (ACh) concentration at the neuromuscular junction (NMJ), causing muscle contraction and eventual paralysis. Mutants with impaired ACh release are resistant to aldicarb-induced paralysis [39]. *egl-30*/Gα_q_ mutants are resistant to aldicarb, whereas *goa-1* mutants are hypersensitive to aldicarb-induced paralysis, indicating that EGL-30/Gα_q_ stimulates and GOA-1/Gα_o_ inhibits ACh release from motor neurons [31]–[33]. Since body wall muscles also receive inhibitory GABA inputs, hypersensitivity to aldicarb can also be caused by decrease in GABA release at the NMJ [40]–[42]. The time course of paralysis of *ser-2* mutants induced by aldicarb was similar to the wild-type (Figure 3A). This may be due to the restricted expression of *ser-2* in a subset of GABAergic neurons or insufficient endogenous tyramine signaling to modulate GABA release under regular assay conditions. We therefore generated transgenic lines that overexpressed *ser-2* in all cholinergic motor neurons (*Pacr-2*::SER-2) or GABAergic (*Punc-47*::SER-2) motor neurons and analyzed the rate of aldicarb-induced paralysis. In the absence of exogenous tyramine, the time course of paralysis of transgenic animals that express SER-2 in cholinergic or GABAergic ventral nerve cord motor neurons was similar to the wild-type (Figure 3A). However, on plates that contained both aldicarb and tyramine, animals that overexpressed *ser-2* in cholinergic neurons (*Pacr-2*::SER-2) were more resistant to the paralytic effects of aldicarb than the wild-type. Conversely, animals that overexpressed *ser-2* in all GABAergic neurons (*Punc-47*::SER-2) were hypersensitive to paralysis on plates containing aldicarb and tyramine (Figure 3B). These data are consistent with the hypothesis that SER-2 couples to the GOA-1/Gα_io_ pathway to inhibit neurotransmitter release.

### Tyramine-Mediated Reduction in GABA Synaptic Release Requires SER-2

**Figure 4:**
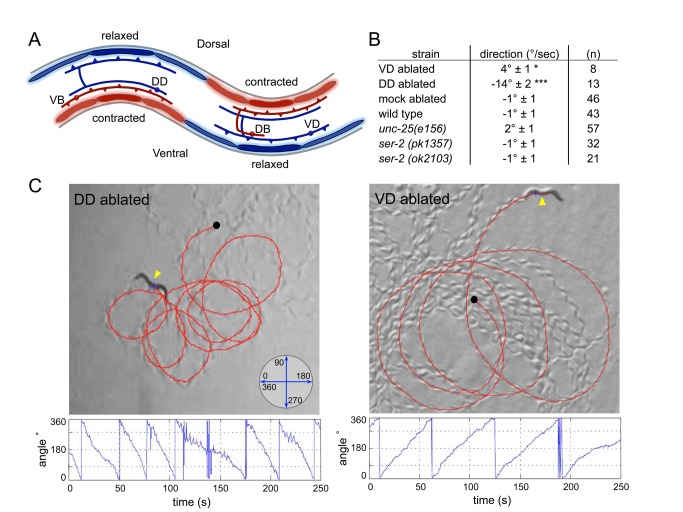
Ablation of VD or DD motor neurons induces a navigational bias. (A) Schematic of D-motor neuron wiring. VD motor neurons receive inputs from cholinergic DB motor neurons, and release GABA on ventral body wall muscles. DD motor neurons receive input from cholinergic VB motor neurons and release GABA on dorsal body wall muscles. Figure adapted from wormatlas.org. (B, C) Killing subsets of GABAergic motor neurons by laser ablation-induced navigational biases. (B) Turning rate (°/sec ± SEM) is affected in animals where VD or DD neurons are ablated. DD-ablated animals navigate with a dorsal bias. VD-ablated animals navigate with a ventral bias. Mock-ablated animals, GABA-deficient mutants (*unc-25*), and *ser-2* mutants did not show a change in turning rate. Turning angle was calculated by worm tracking software; *n* is indicated. (C) Representative locomotory path of a DD-ablated (left panel) and VD-ablated animal (right panel). DD-ablated animals locomote in dorsally directed circles (Movie S1). VD-ablated animals locomote in ventrally directed circles (Movie S2). The direction of locomotion (°) was determined from orientation of the animal's trajectory on the plate (inset). Red line traces the path of locomotion from the origin (black dot); yellow arrow designates the ventral side of the animal. Instantaneous turning angle is plotted for the duration of the locomotion path. Statistical differences calculated from mock ablations: *p<0.05, ***p<0.001, two-tailed Student's t test.

The GABAergic VD motor neurons that express *ser-2* make synaptic contacts onto the ventral musculature (Figure 4A). Therefore, to directly evaluate whether tyramine modulates synaptic release of GABA from motor neurons, we measured the frequency of endogenous inhibitory post-synaptic currents (IPSCs) in whole-cell recordings from ventral body wall muscle cells (Figure 3C–H). To isolate GABA currents, recordings were made from *unc-29; acr-16* double mutants (+/+) that lack excitatory neurotransmission at the NMJ [43],[44]. In *unc-29; acr-16* double mutants, the only remaining currents are mediated through chloride permeation of the GABA_A_-like receptor UNC-49 [43],[45]. In these animals, we observed high levels of endogenous IPSC activity (∼13 events/s) that gradually declined over the time course of the recording period (∼10 min). This basal level of inhibitory activity is consistent with previous reports that have used other, nongenetic approaches to isolate IPSCs. After recording an initial 60 s period of basal activity, we switched to a bath solution containing tyramine. Within 30 s of tyramine exposure we noted a clear decrease in IPSC frequency (Figure 3E,G). The magnitude of this decrease was significantly greater than the slight decrease in IPSC frequency we observed over the same time course in control experiments without tyramine (+ tyramine, 34%±4% decrease; no tyramine, 13%±6% decrease). These results indicate that tyramine can inhibit GABA-mediated transmission at the NMJ. The tyramine-induced reduction was not reversible within the time course of our recordings, which may suggest that tyramine is acting through a high affinity receptor. To test whether the reduction in IPSC frequency involved SER-2, we examined the effects of tyramine exposure in recordings from *unc-29; acr-16; ser-2* triple mutants (Figure 3F,G). The amplitude of endogenous GABA IPSCs was not significantly different between +/+ animals and *ser-2* mutants (+/+, 29.5±1.4 pA; *ser-2*, 31.9±3.9 pA) and was not significantly affected by tyramine exposure for either strain (+/+, 26.9±1.2 pA; *ser-2*, 30.7±4.4 pA), indicating that clustering and function of postsynaptic GABA receptors are normal in *ser-2* mutants, and not affected by tyramine (Figure 3H). The basal IPSC frequency prior to tyramine exposure was also not changed significantly in *ser-2* mutants (+/+, 13.2±1.2 Hz; *ser-2*, 12.5±2 Hz). However, the tyramine-mediated reduction in IPSC frequency we observed in +/+ animals was significantly attenuated in *ser-2* mutant animals (*ser-2*, 17%±4% reduction; +/+, 35%±4% reduction) such that it was indistinguishable from that observed in our control recordings without tyramine (control (no tyramine), 13%±6% reduction). After normalization, tyramine application reduced IPSC frequency by 25%±5% in +/+ compared to 5%±5% reduction in *ser-2* mutants (Figure 3G). Taken together, our data show that tyramine inhibits GABA release onto ventral body wall muscles in a SER-2-dependent manner.

### Ablation of Subsets of GABAergic Motor Neurons Induces a Navigational Bias

*C. elegans* moves on its side by propagating a sinusoidal wave of ventral-dorsal flexures along the length of its body. GABAergic VD motor neurons synapse onto ventral muscles and receive synaptic inputs from DA/DB cholinergic motor neurons that synapse onto dorsal body wall muscles (Figure 4A) [46]. Conversely, GABAergic DD motor neurons synapse onto dorsal muscles and receive synaptic inputs from VA/VB cholinergic motor neurons that synapse onto ventral body wall muscles. This arrangement suggests that a body bend is generated by Ach-mediated muscle contraction on one side and GABA-mediated relaxation on the contralateral side. This hypothesis is supported by the observation that animals in which the VD and DD neurons are killed by laser ablation move with a reduced wave amplitude [46]. The *Pser-2*::GFP and *Pflp-13*::GFP fluorescent markers, which specifically label the 13 VD and 6 DD neurons, respectively, allowed us to further test the role of the VD and DD neurons in laser ablation experiments. Animals in which only the VD neurons were ablated still propagated a sinusoidal wave along the anterior–posterior axis, but displayed deeper ventral than dorsal flexures (Movies S1 and S2). As a consequence VD ablated animals moved in ventrally directed circles (radius 2.20±0.29 body lengths, *n* = 5, Figure 4B,C). Conversely, animals in which the DD neurons were ablated exhibited deeper dorsal than ventral flexures and moved in dorsally directed circles (radius 0.61±0.11 body lengths, *n* = 10). GABA-deficient *unc-25* mutants made shallow body bends, but showed no directional bias in their locomotion pattern. Thus, the specific ablation of the VD or DD GABAergic neurons indicate that asymmetric relaxation of either the ventral or dorsal body wall muscles results in a directional bias in locomotion.

### Induction of Turning Behavior Through Optogenetic Control of GABAergic Motor Neurons

**Figure 5:**
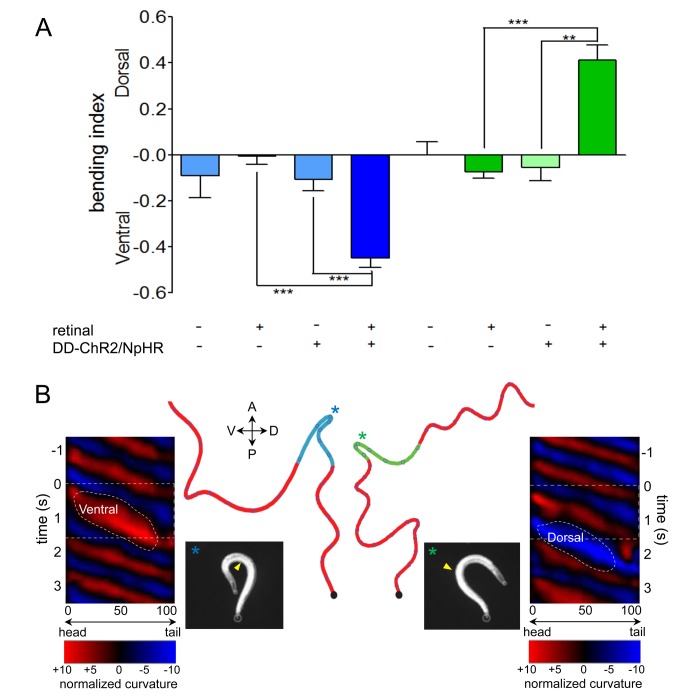
Optogenetic control of navigation. (A, B) Acute modulation of the activity of DD GABA neurons in transgenic animals expressing channelrhodopsin (ChR2) and halorhodopsin (NpHR) in DD motor neurons (*Pflp-13*::ChR2::GFP; *Pflp-13*::NpHR::CFP) induces turning behavior. Blue light activation of GABAergic DD motor neurons that synapse onto dorsal muscles induces ventral turning. Green light inhibition of GABAergic DD motor neurons induces dorsal turning. (A) Quantification of bending behavior with green and blue light exposure. Bending bias was calculated as the fraction of dorsal turns – fraction of ventral turns after blue (DD activation, blue bars) or green (DD inhibition, green bars) light exposure. Each bar represents the mean bending bias for a minimum of 45 animals per genotype. Statistical significance as indicated: **p<0.001 and ***p<0.0001, two-tailed Student's t test. (B) Locomotion traces signify the time course of blue and green light exposure during forward movement (red). Open circles denote 1-s time marks. The compass indicates anterior (A), posterior (P), ventral (V), and dorsal (D) directions. Kymographs display sinusoidal bending wave amplitude before, during, and after light exposure. Normalized curvature is plotted at each point along the worm's centerline in units of inverse worm lengths. Color indicates curvature in either the ventral (red) or dorsal (blue) direction. The colored bands widen and brighten during deep turns induced by light exposure: white horizontal dotted lines indicate duration of light exposure, Ventral indicates a deep ventral bend, and Dorsal indicates a deep dorsal bend. Still images were taken at * location on worm track (Movie S3). Yellow triangle indicates the position of the vulva.

Our data suggest that the modulation of the activity of either the VD or DD motor neurons allows the animal to bend and steer in either a ventral or dorsal direction. To determine if acute activation or inhibition of a specific subclass of GABAergic neurons could induce turning behavior, we used optogenetic stimulation and inhibition. The complexity of the *ser-2* promoter, which contains coding sequences and alternative start sites, did not allow us to highly express light-activated channels in the VD neurons. To test whether the differential activity of the VD and DD motor neurons can induce bending, we generated transgenic animals that co-expressed the light-activated cation channel Channelrhodopsin-2 (ChR2) [47] and light-activated chloride pump Halorhodopsin (NpHR) [48] in the six GABAergic DD motor neurons that synapse onto the dorsal muscles (*Pflp-13*::ChR2; *Pflp-13*::NpHR). ChR2 is activated by blue light and depolarizes neurons, while NpHR is activated by green light and hyperpolarizes neurons. We found that blue light activation induced a deep ventral turn (Figure 5 and Movie S3). In contrast, green light inhibition induced a deep dorsal turn. This turning behavior was not observed in nontransgenic animals or in transgenic animals raised on plates without all-*trans*-retinal, the chromophore of ChR2 and NpHR. To quantify turning behavior we calculated a bending index as the fraction of animals that turned ventrally or dorsally in response to light exposure (Figure 5A). A bending index of zero indicates no directional bias, whereas a negative or positive fraction indicates a ventral or dorsal bias, respectively. The bending index of *Pflp-13*::ChR2/NpHR transgenic animals was −0.45±0.04 with exposure to blue light and 0.41±0.06 for animals with exposure to green light. The *flp-13* promoter also drives expression in a small set of head neurons in addition to the DD neurons. To determine if the activation or inhibition of the DD neurons was sufficient to induce bending, we used an optogenetic illumination system capable of tracking and stimulating individual regions of a freely moving animal [49]. Targeted illumination of the animal's ventral nerve cord that harbors the DD neuronal cell bodies induced a tight ventral bend in response to blue light activation and a tight dorsal bend in response to green light inhibition (Figure 5B and Movie S3). Switching between blue and green light exposure enabled the remote control of ventral and dorsal turning behavior in freely moving *Pflp-13*::ChR2/NpHR transgenic animals. Thus, the acute stimulation of GABA release on the dorsal side induced relaxation of the dorsal muscles resulting in a ventral turn. Conversely, the acute inhibition of GABA release onto the dorsal muscles resulted in hypercontraction of dorsal muscles and a dorsal bend. Our data indicate that modulating the activity of subsets of GABAergic neurons synapsing onto either the dorsal (DD) or ventral side (VD) of the animal can induce navigational bias.

### *ser-2* Facilitates the Execution of Omega Turns in the Escape Response

**Figure 6:**
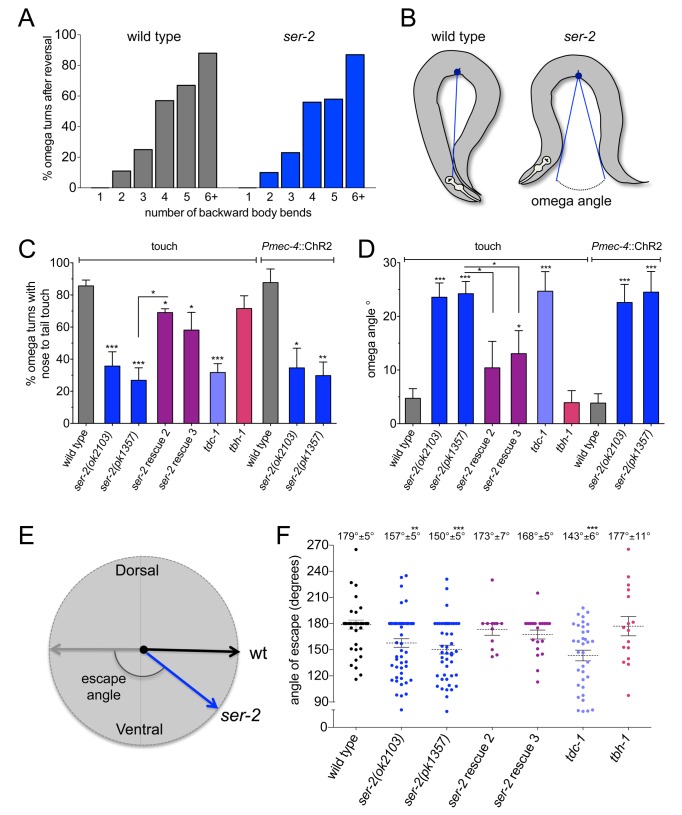
*ser-2* mutants make shallow omega bends. (A) Distribution of touch-induced reversals ending in an omega turn. Omega turns are more likely to occur after longer reversals (>3 body bends). Wild-type and *ser-2* mutant animals initiate omega turns at the same rate (*n*≥150 per genotype). (B) Schematic representation of the omega angle. The omega angle was measured as the angle from the deepest point in the ventral bend to the closest points anterior and posterior of the animal. Images were adapted from movies of animals in the most ventrally contracted state of the escape response. (C) Percent of omega turns where the animal's nose touches the tail during the execution of the turn (closed omega turn). *ser-2* mutants [*ser-2(ok2103)*, *n* = 52; *ser-2(pk1357)*, *n* = 6 2] touch nose to tail less frequently than wild-type (*n* = 51) in omega turns induced by both touch (Movies S4 and S5) and blue light in a *Pmec-4*::ChR2 background [*Pmec-4*::ChR2, *n* = 38; *ser-2(ok2103)*; *Pmec-4*::ChR2, *n* = 43; *ser-2(pk1357)*; *Pmec-4*::ChR2, *n* = 28]. Tyramine/octopamine-deficient *tdc-1* mutants touch nose to tail less frequently than wild-type [*tdc-1(n3420)*, *n* = 144], while octopamine-deficient *tbh-1* mutants close omega turns like the wild-type [*tbh-1(n3247)*, *n* = 153]. Genomic rescue lines partially restore this omega turning defect (*ser-2* rescue line 2, *n* = 20; *ser-2* rescue line 3, *n* = 21). (D) Average omega angle measured after touch or exposure to blue light in a *Pmec-4*::ChR2 background [*Pmec-4*::ChR2, *n* = 38; *Pmec-4*::ChR2; *ser-2(ok2103)*, *n* = 43; *Pmec-4*::ChR2; *ser-2(pk2103)*, *n* =  28]. *ser-2* mutants [*ser-2(ok2103)*, *n = *52; *ser-2(pk1357)*, *n* = 62] and tyramine/octopamine-deficient mutants [*tdc-1(n3420)*, *n = *35] make a wider omega turn than wild-type (*n* = 51). Octopamine-deficient *tbh-1* mutants do not make wider omega turns [*tbh-1(n3247)*, *n* = 16]. Genomic rescue lines partially restore the omega angle defect of the mutants (*ser-2* rescue line 2, *n* = 20; *ser-2* rescue line 3, *n* = 21). (E) Escape angles were measured from the direction of the reversal (induced by gentle anterior touch) to the direction of reinitiated forward locomotion. (F) Distribution of escape angles. Dashed grey line indicates average. Wild-type animals and *tbh-1* mutants escape in the opposite direction from the touch stimulus [wt, 179°±5°, *n* = 42; *tbh-1(n3427)*, 177°±11°, *n* = 16]. *ser-2* mutants and *tdc-1* mutants make a shallower escape angle [*ser-2 (ok2103)*, 157.5°±5°, *n* = 53; *ser-2(pk1357)*, 150°±5°, *n* = 46; *tdc-1(n3420)*, 143.3°±6°, *n = *35]. Genomic rescue lines restore the escape angle to wild-type levels (*ser-2* rescue line 2, 173°±7°, *n* = 12; *ser-2* rescue line 3, 168.5°±5°, *n* = 20). Rescue denotes the transgenic line *Pser-2*::SER-2; *ser-2(pk1357).* Error bars depict SEM. Statistical differences calculated from wild-type unless otherwise indicated: *p<0.05, **p<0.01, ***p<0.001, two-tailed Student's *t* test.

**Figure 7:**
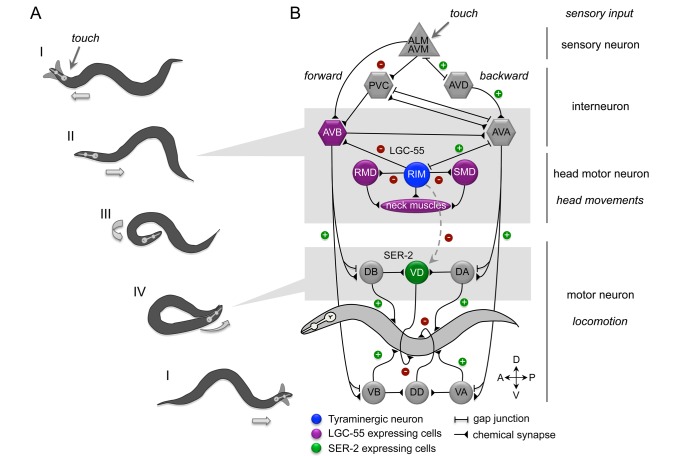
Model: Tyramine orchestrates the *C. elegans* escape response through the activation of ionotropic and metabotropic receptors. (A) Silhouettes of the four phases of the *C. elegans* anterior touch escape response. Images were adapted from a movie of an animal executing an escape response. See text for details. (B) Schematic representation of the neural circuit that controls the *C. elegans* escape response. Synaptic connections (triangles) and gap junctions (bars) are as described by White et al. (1986) [20]. Green plus signs represent excitatory connections, and red minus signs indicate inhibitory connections. Sensory neurons are shown as triangles, command neurons required for locomotion are hexagons, and motor neurons are depicted as circles. The compass indicates anterior (A), posterior (P), ventral (V), and dorsal (D) directions. *C. elegans* sinusoidal locomotion is propagated by alternatively contracting and relaxing opposing ventral and dorsal body wall muscles of the animal using cholinergic (DB and VB for forward and DA and VA for backward locomotion) and GABAergic (VD and DD) motor neurons. Anterior touch induces the activation of the tyramine release from the RIM motor neurons (blue cells). Solid lines represent synaptic activation of LGC-55 in neurons and muscles (purple cells) that result in the inhibition of forward locomotion and suppression of head movement in the initial phase of the escape response. Dashed lines represent extrasynaptic activation of SER-2 in the GABAergic VD motor neurons (green cells). The activation of SER-2 causes a decrease in GABA release on the ventral side animal. This allows the hypercontraction of muscles on the ventral side of the animal, thus facilitating the execution of a ventral omega turn.

The expression of *ser-2* in the GABAergic VD neurons suggested a possible role in *C. elegans* navigation. However, *ser-2* mutants moved normally and did not display a ventral or dorsal directional bias (Figure 4B) possibly through a lack of basal tyramine to activate SER-2 during regular locomotion. We have previously shown that tyramine coordinates the timing of backing locomotion and the suppression of head movements in the escape response [13]. Gentle anterior touch triggers tyramine release from the RIM neurons and the synaptic activation of the tyramine-gated chloride channel, LGC-55 [22]. Is SER-2 also required in the execution of the escape response? Touch-induced reversals are often coupled to a sharp omega turn, which allows the animal to change locomotion in a direction opposite to its original course (Figures 6E and 7A) [18]. The omega turn is initiated by a steep ventral bend of the head when the animal reinitiates forward locomotion. While the sharp bend is propagated posteriorly along the body, the head usually slides along the ventral side of the body. We analyzed turning behavior in response to gentle anterior touch. We found that the likelihood of engaging in an omega turn (>90° turn initiated by the first forward head swing) was correlated with the length of the reversal (Figure 6A). Short reversals most often resulted in shallow head bends and modest deflections from the original trajectory. In contrast, escape responses that included reversals of four or more body bends most often ended in an omega turn. These results are consistent with previous studies of reversals where omega turns tend to occur after a long reversal [50]–[52].

Omega turns that occurred in response to anterior touch were exclusively made on the ventral side of the animal (*n*≥250). In response to touch, animals in which the GABAergic DD neurons were ablated initiated omega turns with a deep ventral head bend, but often their head failed to touch the ventral side of the body to close the omega turn (Figure S5). Animals with ablated GABAergic VD neurons did not have a defect in the execution of closed omega turns. The initiation of the omega turn may be triggered by the RIV head motorneurons that innervate ventral neck muscles [51]. The failure of DD ablated animals to fully close their omega turns indicates that the propagation of a sharp bend along the body requires the hypercontraction of ventral muscles and relaxation of dorsal muscles.

Tyramine-deficient *tdc-1* mutants or RIM ablated fail to suppress head movements, make short reversals, and less frequently engage in the execution of omega turns [13],[51]. In contrast, in response to anterior touch, *ser-2* mutants suppressed head movements and reversed similar to wild-type animals (Figure S6). Furthermore, *ser-2* mutants initiated an omega turn with a steep ventral head bend at the same frequency as the wild-type (Figure 6A). However, once *ser-2* mutants initiated the omega turn, the ventral turn was less deep than the wild-type. Whereas most wild-type animals' heads touched the ventral side of the body (86%±3.6%, *n* = 51) during an omega turn, *ser-2* mutant animals failed to fully close the omega turn (27%±7.8%, *n* = 62) (Figure 6B,C and Movies S4 and S5). Like the *ser-2* mutants, *tdc-1* mutants that are unable to synthesize tyramine and octopamine [13] failed to fully close the omega turn during the escape response (*tdc-1* 32%±5.5%, *n* = 144). In contrast, *tbh-1* mutants, which only lack octopamine, executed closed omega turns (*tbh-1* 72%±7.9%, *n* = 153), comparable to the wild-type. We measured the angle of the omega turn (omega angle) from the deepest most contracted region of the body to the closest or touching points in the head and tail (Figure 6B,D). Wild-type animals typically fully closed their omega bend, while *ser-2* mutants often failed to close omega bends, averaging an omega angle of 24°±2.3° (*n* = 62). Animals lacking tyramine and octopamine (*tdc-1)* averaged an omega angle of 25°±3.6°, while animals lacking octopamine (*tbh-1)* alone closed their omega turn. Since touch stimuli in these assays have some inherent variability, we also induced reversals by optogenetic activation of the touch sensory neurons. Light induces an escape response in *Pmec-4*::ChR2 transgenic animals that express the ChR2 in the touch sensory neurons [47],[49],[53]. We analyzed omega turns of *Pmec-4*::ChR2 transgenic animals in response to blue light in both wild-type and *ser-2* mutant backgrounds. The light-induced escape response of *ser-2* mutants showed a similar defect in omega turns as with reversals induced by touch and had a lower frequency of closed omega turns than the wild-type (Figure 6C,D). The turning defects of *ser-2* mutants caused an alteration in the direction of reinitiated forward movement, or escape angle. In response to touch, wild-type animals and *tbh-1* mutants completely reversed their direction of locomotion with an escape angle of 179°±5° (*n* = 42) and 177°±11° (*n* = 16), respectively. In contrast, in response to touch, *ser-2* mutants and *tdc-1* mutants made a more shallow escape angle, changing their direction from the point of stimulus by 150°±5° (*n* = 46) and 143°±6° (*n* = 35) (Figure 6E,F). Genomic rescue lines partially restore the omega angle defect of the mutants and restored the escape angle to wild-type levels. Our data indicate that tyraminergic activation of SER-2 facilitates the execution of a tight ventral bend in the escape.

## Discussion

The concept of monoaminergic coding of behaviors originated from work in crustaceans and insects where fully coordinated behavioral sequences can be elicited by the injections of specific monoamines into the nervous system [6],[16],[17]. However, understanding how sensory inputs recruit the action of monoamines and how changes in circuit properties affect behavior remains a tremendous challenge in the mammalian and even insect nervous systems. Tyramine release from a single pair of neurons, acting on few tyramine receptors in the *C. elegans* nervous system with single-cell resolution, provides unique insights into how monoamines orchestrate independent motor programs in a complex behavior. In this study we analyzed how the G-protein coupled receptor SER-2 modulates the output of a neural circuit in a compound motor sequence.

### Tyramine Inhibits GABA Release

Our genetic data suggest that the tyramine receptor SER-2 acts in a Gα_o_ (GOA-1) pathway. The Gα_o_/GOA-1 is expressed in all *C. elegans* neurons where it antagonizes Gα_q_/EGL-30 function in many behaviors including locomotion, egg laying, and pharyngeal pumping [54],[55]. Dopaminergic and serotonergic G-protein coupled receptors are expressed in *C. elegans* ventral cord neurons and their hyperactivation with exogenous dopamine or serotonin can induce paralysis within minutes [56]–[59]. However, *ser-2* and other individual biogenic amine receptor mutants have no obvious locomotion defects. This indicates that Gα_o_/GOA-1 and Gα_q_/EGL-30 integrate monaminergic signals to modulate neurotransmitter release from the motor neurons to control locomotion (Figure 1B). Dopamine, serotonin, and tyramine may allow the animal to refine locomotory patterns during different behavioral states. Gα_o_/GOA-1 activity is thought to reduce the abundance of the synaptic priming protein UNC-13 at the synapse in *C. elegans* ventral cord neurons [33]. Our data indicate that tyramine reduces GABA release from VD motor neurons in a SER-2-dependent manner to augment the reorientation component of the escape response. In this regard, SER-2 shares similarities with mammalian alpha(2)-adrenergic receptors that inhibit neurotransmitter release and cause vasoconstriction during a fight-or-flight response [4].

### Tyramine Acts Through Synaptic Activation of Ionotropic Receptors and Extrasynaptic Activation of Metabotropic Receptors

Tyramine can act as a classical neurotransmitter in *C. elegans* through the synaptic activation of the tyramine-gated chloride channel, LGC-55 [22],[60]. While LGC-55 is predominantly expressed in cells that are directly synaptic to tyraminergic RIM neurons, SER-2-expressing cells do not receive direct RIM innervation, indicating that SER-2 activation occurs extrasynaptically [28],[36]. In addition, ionotropic and metabotropic receptors have distinct ligand affinity and signaling kinetics. Ligand-gated ion channels like LGC-55 have a relatively low affinity for their ligand (Kd 0.1 to 1 mM) and affect postsynaptic potentials within milliseconds. This allows for fast localized signaling between neurons and their postsynaptic partners. In contrast, G-protein coupled receptors have a high affinity for their ligand (Kd 0.1–1 µM) and operate on timescales from seconds to minutes [61]. Synaptic spillover from the synaptic cleft and diffusion can activate these high affinity receptors that are distant from the release site. As tyramine is released from a single pair of head neurons that extend processes into the nerve ring, the activation of SER-2 in the GABAergic VD neurons depends upon the diffusion of tyramine through the pseudocoelomic space to reach the VD processes along the length of the body. *C. elegans* has two other G-protein coupled receptors, TYRA-2 and TYRA-3, in addition to SER-2 that bind tyramine with high affinity [26],[27]. *tyra-2 and tyra-3* are not expressed in cells that receive direct synaptic inputs from the tyraminergic RIM neurons [62]. Furthermore, no tyramine or octopamine reuptake transporter has been identified in either *C. elegans* or *Drosophila*, which suggests that tyramine diffusion from the synaptic cleft is part of its mechanism of action. Serotonin, dopamine, and octopamine receptors are also expressed in many *C. elegans* cells that are not directly postsynaptic to the small number of monoaminergic neurons that release them [36],[58]. Similarly, in humans the monoaminergic cells are grouped in relative small nuclei that can affect large areas of the CNS or the periphery that do not receive direct synaptic inputs. Thus, monoamines are not confined to the anatomical connectome and can reconfigure outputs to large neuronal ensembles [63].

### Tyramine Temporally Coordinates Independent Motor Programs in a Complex Behavior

The translation of sensory input into goal-directed behaviors requires the temporal coordination of independent motor programs. In response to gentle touch to the head, *C. elegans* engages in a compound motor sequence that allows the animal to retreat and navigate away from a touch stimulus (Figure 7A). In the base state, (I) forward locomotion is accompanied by exploratory head movements in which the tip of the nose moves from side to side. Gentle touch to the head of *C. elegans* elicits an escape response where the animal reverses its direction of locomotion while suppressing its exploratory head movements (II). Classic laser ablation experiments by Chalfie et al. (1985) [19] complemented by the known neural wiring diagram support a model in which the touch sensory neurons (ALM/AVM) inhibit the forward locomotion “command” neurons (PVC/AVB) and activate the backward locomotion “command” neurons (AVD/AVA), causing the animal to move backward away from the stimulus (Figure 7B). The AVA backward locomotion “command” neurons activate the electrically coupled tyraminergic RIM motor neurons, which exhibit coactivated calcium transients with the AVA [64],[65]. Tyramine release from the RIM induces the suppression of head movements through the activation of the fast-acting inhibitory chloride channel, LGC-55, in neck muscles and cholinergic motor neurons that control head movements [13],[22]. Activation of LGC-55 in the AVB forward locomotion “command” neurons further inhibits forward locomotion, stimulating long reversals that are coupled to the initiation of an omega turn [50],[51]. The reversal is followed by a deep ventral head bend (III), allowing the animal to make a sharp (omega) turn (IV) where the head of the animal slides along the ventral side of the body and resumes forward locomotion (I). Our data show that extrasynaptic activation of the G-protein coupled tyramine receptor SER-2 contributes to the proper execution of the omega turn during the last stage of the escape response. The RIV and SMD head motor neurons have been implicated in the ventral head bend that initiates the omega turn [51]. *ser-2* mutants properly initiate the turn but bend less deep and the head often fails to touch the tail during the omega turn (IV).

*C. elegans* locomotion is controlled by cholinergic (A and B) and GABAergic (D) motor neurons that propel a wave of ventral-dorsal muscle flexures along the length of the body. We show that SER-2 inhibits neurotransmitter release from the GABAergic VD neurons. VD motor neurons release GABA on ventral body wall muscles and receive inputs from cholinergic DB neurons that synapse onto dorsal muscles. The DD motor neurons receive input from cholinergic VB motor neurons and release GABA on dorsal body wall muscles (Figure 4A) [20]. This organization indicates that GABAergic motor neurons are essential for contralateral muscle relaxation, thus promoting local bending during wave propagation [46].

While spatial orientation in response to sensory stimuli is a fundamental component of animal behavior, the neural correlates are poorly understood. To change its direction of movement and navigate its environment, an animal needs to generate asymmetry in its locomotion pattern [66],[67]. We show that the ablation of GABAergic VD neurons induces ventrally directed movement likely through the failure to properly relax the ventral muscles. Conversely, ablation of GABAergic DD neurons induces dorsally directed movement. Our experiments provide functional evidence that VD and DD neurons inhibit ventral and dorsal bending. Strikingly, the acute inhibition or activation of the DD motor neurons induces a sharp dorsal or ventral turn, respectively. Optogenetic neuronal stimulation can elicit distinct motor responses in multiple systems [47],[68]–[70]. The optogenetic control of a single class of GABAergic neurons induces bi-directional steering and the remote control of animal navigation.

The ventral omega turn in the escape response occurs several seconds after the touch-induced reversal, which is within the time scale that GPCRs modulate neuronal activity. To make a tight turn the animal needs to generate an asymmetry in the excitation and inhibition of the ventral and dorsal muscle quadrants. Our data indicate that SER-2-mediated inhibition of GABAergic release onto the ventral muscles stimulates ventral muscle contraction and the proper propagation of the ventral body bend during the omega turn (Figure 7B). The kinetics of synaptic activation of the ion channel LGC-55 and extrasynaptic activation of the G-protein coupled receptor SER-2 temporally coordinates different phases of the escape response. Interestingly, the tyramine GPCR TYRA-3 modulates responses to pain-like stimuli and decision making [27],[62]. This indicates that tyramine, much like the mammalian adrenergic signaling, coordinates different aspects of a flight response.

Our results provide molecular and neural insights on how monoamines reconfigure the output of neural circuits and orchestrate complex behaviors. Many neurotransmitters, including serotonin, GABA, acetylcholine, and glutamate, act through both ion channels and GPCRs in animals ranging from worms to man. We hypothesize that the temporally coordinated activation of ionotropic and metabotropic receptors may be a common signaling motif employed across organisms to orchestrate behavioral responses. Furthermore, the different monoamines appear to play a strikingly conserved role in the specification of distinct internal states, suggesting that the principles of neuronal modulation are conserved across species.

## Materials and Methods

All *C. elegans* strains were grown at room temperature (22°C) on nematode growth media (NGM) agar plates with OP50 *E. coli* as a food source [71]. The wild-type strain used in this study was Bristol N2. G-protein coupled receptor and signaling component mutants used in this study were OH313: *ser-2(pk1357)*, QW329: *ser-2(ok2103)*, QW245: *lgc-55(tm2913)*, QW42: *tyra-2(tm1815)*, VC125: *tyra-3(ok325)*, JT734: *goa-1(sa734)*, JT748: *dgk-1(sa748)*, JT609: *eat-16(sa609)*, EG4532: *egl-30(tg26)*, CB156: *unc-25(e156)*, CB151: *unc-3(e151)*, QW284: *tdc-1(n3420)*, MT9455: *tbh-1(n3247)*, QW542: *unc-25(e156); ser-2(pk1357)*, QW40: *lgc-55(n4331); unc-3(e151)*, QW41: *ser-2(pk1357) unc-3(e151)*, QW837*: lgc-55(tm2913); ser-2(pk1357)*, and QW838*: lgc-55(tm2913); ser-2(pk1357) unc-3(e151)*. The rescue strains used were QW198: [*Pser-2*::SER-2::GFP, *lin-15(+)*]*(zfEx34); ser-2(pk1357) lin-15(n765ts)*, QW411: [*Pser-2*::SER-2::GFP, *lin-15(+)*]*(zfEx70); ser-2(pk1357) lin-15(n765ts)*, QW412: [*Pser-2*::SER-2::GFP, *lin-15(+)*]*(zfEx301); ser-2(pk1357) lin-15(n765ts)*, QW196: [*Punc-47*::SER-2, *lin-15(+)*]*(zfEx33)*; *ser-2(pk1357) lin-15(n765ts)*, QW897: [*Punc-47*::GOA-1::SL2::mCherry, *unc-122*::GFP] (*zfEx347*); *goa-1(sa734)*, QW895: [*Punc-47*::EAT-16::SL2::mCherry, *unc-122*::GFP] (*zfEx346*); *eat-16(sa609)*, and QW194: [*Pacr-2*::SER-2, *lin-15(+)*]*(zfEx32); ser-2(pk1357) lin-15(n765ts).* The strains used for cell identification were EG1285: *Punc-47*::GFP*(oxIs12)*, CX2835: *Pglr-1::GFP(kyIs29)*, LX929: *Punc-17*::GFP*(vsIs48)*, NY2037: *Pflp-13*::GFP*(ynIs37)*, OH2246: *Pser-2::GFP(otIs107)*, QW192: *Pser-2*::mCherry*(zfIs8)*, QW122: *Plgc-55*::GFP*(zfIs6)*, and QW84: *Plgc-55*::mCherry*(zfIs4).* The strains used for electrophysiological analysis were IZ33*: unc-29(x29); acr-16(ok789)* and *IZ598: unc-29(x29); acr-16(ok789); ser-2(pk1357).* The strains used for optogenetic assays were QW410: [*Pmec-4*::ChR2::YFP, *lin-15(+)*]*(zfIs18); ser-2(pk1357)*, QW409: [*Pmec-4*::ChR2::YFP, *lin-15(+)*]*(zfIs18); ser-2(ok2103)*, and QW429: [*Pflp-13*::ChR2::GFP*; Pflp-13*::NpHR::CFP, *lin-15(+)*]*(zfIs32); lite-1(ce314).*

Transgenic strains were generated by microinjection of plasmid DNA into the germ line of *lin-15(n765ts*) mutants with the pL15EK rescuing plasmid. Extrachromosomal arrays were integrated by X-ray irradiation (120 kV) and resulting transgenic strains were outcrossed at least four times to N2. A *Pser-2*::SER-2::GFP rescue construct was made by cloning 10.2 kb of genomic sequence including 2.2 kb upstream of the first translational start site into the pPD95.70 vector. The genomic rescue constructs used for exogenous tyramine and omega turn assays were injected between 10 and 20 ng/µl. A *Pser-2*::mCherry mini-gene reporter was constructed by cloning an 11.8 kb sequence that included the three start sites, first intron, and part of exon 2 into the pDM1247 vector. The GABAergic and cholinergic cell-specific rescue lines were cloned using *ser-2a* cDNA behind the *unc-47* (1.2 kb) [72] and *acr-2* (3.4 kb) promoters, respectively [73].

A *Pmec-4*::ChR2::YFP plasmid [47] was injected at 80 ng/µl. The integrated strain was crossed into two *ser-2* mutant allele backgrounds. *Pflp-13*::ChR2::GFP; *Pflp-13*::NpHR::CFP was cloned by inserting a 2 kb promoter from *Pflp-13*::GFP [37] into ChR2 and NpHR vectors [47],[48]. The integrated strains carrying ChR2 or NpHR transgenes were cultured on NGM agar plates containing OP50 *E. coli* supplemented with 1.3 mM all-*trans*-retinal for one generation. Larval L4 animals were transferred to retinal plates 24 h before behavioral assays.

Ventral nerve cord expression analysis was performed using *Pser-2*::mCherry, *Punc-47*::GFP, and *Punc-17*::GFP transgenic animals. Head muscle analysis was done using *Pser-2::GFP* and *Plgc-55*::mCherry. Images were taken using confocal microscopy (Zeiss and Pascal imaging software) and formatted using ImageJ software.

### Behavioral Assays

Behavioral assays were performed at room temperature. Drug assays were conducted on young adult animals aged 24 h post-L4 larval stage. Locomotion assays were performed on agar plates containing 2 mM acetic acid with or without tyramine hydrochloride (Sigma-Aldrich). Approximately 10 animals were transferred to assay plates and scored for locomotion every minute over a 20-minute period. Animals were scored as immobilized if there was no sustained forward or backward locomotion in a 5-s interval. Aldicarb drug assays were performed using NGM agar plates supplemented with 0.5 mM aldicarb (Sigma-Aldrich) with or without 30 mM tyramine. Locomotion is not obviously affected on plates with 30 mM tyramine dissolved in NGM agar instead of the agar used in exogenous tyramine paralysis assays. Animals were scored as paralyzed when they did not move when prodded with a platinum wire.

Optogenetic blue and green light-induced bending assays were performed with *Pflp-13*::ChR2::GFP; *Pflp-13*::NpHR::CFP; *lite-1(ce314)* transgenic animals. Bending behavior movies, worm tracking traces, and kymographs were generated using the CoLBeRT worm tracking system as previously described [49]. Animals were placed in between two glass slides in 200 µl of NGM containing 30% dextran. The space between the slides was approximately 0.127 mm and limited locomotion to two dimensions. The ventral nerve cord was illuminated with blue or green light using the micromirror control of the CoLBeRT system, and the behavior was analyzed using custom tracking software written in MATLAB. For assays used to calculate a bending index, young adult animals were transferred to NGM plates without food for 45 min. Food deprivation stimulated long forward runs, which facilitated the analysis of light-induced bending. Animals were exposed to blue or green light, using GFP (525 nm) and Rhodamine (550 nm) filters for 3 s. A dorsal or ventral bend were scored if the bend was larger than 45°. The bending index was calculated as the fraction of dorsally bending worms minus the fraction of ventrally bending worms. A positive fraction indicates a dorsal bias, a negative fraction indicates a ventral bias, and a zero value represents no directional bias or no response to light exposures.

Omega turns were analyzed on NGM agar plates 2 d postpouring to control for assay plate humidity. Assay plates (60 mm diameter) were seeded with 40 µl OP50 *E. coli* and grown overnight at 37°C to produce a thin bacterial lawn. Young adult worms were transferred to an omega assay plate and allowed to acclimate for at least 10 min. Omega turns were induced by gentle anterior touch with fine eyebrow hair while recording using a FireWire camera and Astro IIDC software. For *Pmec-4*::ChR2::YFP-induced omega turns, animals were exposed to blue light for 5 s to induce a reversal. Bending angles and locomotion trajectories were calculated using Image J software analysis of movie stills. An omega turn was classified as a sharp turn larger than 90° from the initial trajectory, following a reversal of three or more body bends. The omega angle was measured using the deepest part of the bend as the apex with vectors extending to the closest points along the body. Angles larger than 60° were not scored. The escape angle was measured as the angle between the reversal trajectory and the trajectory of reinitiation of forward locomotion after the omega turn.

### Laser Ablations

Animals were mounted on agar pads and anesthetized with 20 mM sodium azide. Laser ablations were done using standard methods [74]. DD and VD motor neurons were identified in *Pflp-13*::GFP animals in the L2 larval stage and *Pser-2*::GFP animals in the L3–L4 larval stage, respectively. Following a recovery period of 1 to 3 d postablation, locomotion and omega turn assays were conducted on young adult animals. Locomotion patterns of animals that exhibited coordinated long runs were recorded at 7.5 fps. Movie analysis was done using MATLAB and the MATLAB Image Acquisition Toolbox [75]. To determine directionality for each locomotion trace, the slope of instantaneous direction over time was measured for individual 360 degree turning events. Laser ablation of motor neurons was confirmed by lack of GFP expression in the cell body positions in adult animals following behavioral experiments.

### Electrophysiology

Endogenous postsynaptic currents were recorded from body wall muscles as previously described [43]. All electrophysiology experiments were carried out at room temperature. Adult animals held at drop of bath solution were glued down to the sylgard coated glass coverslip with cyanoacrylate tissue adhesive (Skinstitch Corp.) applied along the dorsal side of the body. A longitudinal incision was made by sharp glass electrode tip in the dorsolateral area, the intestine and gonad were removed, and the cuticle flap along the incision was glued down in order to expose the ventral medial body wall muscles along the ventral nerve cord. The preparation was then washed briefly for ∼20 s with a solution of collagenase type IV from Clostridium hystolyticum (Sigma-Aldrich) in extracellular bath solution (at a concentration of 1 mg/ml) in order to remove the basement membrane overlying the muscles.

The extracellular solution consisted of 150 mM NaCl, 5 mM KCl, 4 mM MgCl_2_, 1 mM CaCl_2_, 15 mM HEPES, and 10 mM glucose (pH 7.4, osmolarity adjusted with 20 mM sucrose). The intracellular fluid (ICF) consisted of 25 mM K-gluconate, 115 mM KCl, 0.1 mM CaCl_2_, 50 mM HEPES, 5 mM Mg-ATP, 0.5 mM Na-GTP, 0.5 mM cGMP, 0.5 mM cAMP, and 1 mM BAPTA (pH 7.4, osmolarity adjusted with 10 mM sucrose). Whole-cell voltage clamp recordings from *C. elegans* body wall muscle cells (group of ventral medial muscles 9, 11, 13) from +/+ and *ser-2* mutant strains were performed as previously described [43] using an EPC-10 amplifier (HEKA). Data acquisition and voltage protocols were controlled by HEKA Patchmaster software. Patch-clamp electrodes were pulled from borosilicate glass with filament (Sutter Instrument), fire-polished to a resistance of 4–6 MΩ, and filled with an internal solution. 1 M KCl electrode with agarose bridge served as reference electrode. Leak currents and liquid junction potentials were not compensated, though pipette offset was zeroed immediately before getting a gigaohm seal. The membrane potential was clamped at −60 mV. Data were digitized at 6.67 kHz and low pass filtered at 3.3 kHz. Membrane capacitance and the series resistance (at least 20%, up to 60%) were compensated, and only recordings in which the series resistance was stable throughout the course of the recording were included. Endogenous synaptic activity data were collected in continuous mode (saved as 30-s recording sweeps). Typically, 60–90 s of control endogenous activity was recorded followed by a 90-s bath tyramine application (100 µM), and subsequent tyramine wash-out (occasional). The muscle preparation was continuously perfused by extracellular solution with or without tyramine by gravity-flow at a perfusion rate of 2 ml/min. Cells were excluded from analysis if a leak current >300 pA was observed. Only recordings with series resistance R_s_<15 MΩ were included in the analysis. Data analysis and graphing were performed using Excel (Microsoft), Igor Pro (WaveMetrics Inc.), and GraphPrism (GraphPad Software). Mini Analysis software (Synaptosoft Inc.) was used to detect and analyze the endogenous events off-line. Parameters for detection of events were set as follows: amplitude threshold 12 pA, period to search local minimum 80 ms, time before a peak for baseline 20 ms, period to search a decay time 30 ms, fraction of peak to find a decay time 0.37, period to average a baseline 10 ms, area threshold 10, and number of points to average for peak 3. In addition, following automatic detection of endogenous postsynaptic currents, traces were visually inspected, and all events were manually verified and accepted/rejected or recalculated, if necessary. Sixty seconds of continuous data were used in analysis. Segments of 60-s recording prior to tyramine application served as a control. The tyramine effect was evaluated in the last 60 s of a 90-s perfusion—that is, initial 30 s of recording with tyramine in bath solution were eliminated from analysis in order to allow enough time for adequate tyramine perfusion and stabilization effect. Data from each group were averaged, and statistical significance between the strains was determined by two-tailed unpaired Student's *t* test. All chemicals were purchased from Sigma-Aldrich.

## Supporting Information

Figure S1 *C. elegans* become immobilized on exogenous tyramine in a dose-dependent manner. (A) Wild-type animals become immobilized within 5 min on 30 mM tyramine (also see Pirri et al., 2009 [22]). (B) *ser-2* mutants are resistant to body immobilization compared to wild-type, but become immobilized by 60 mM tyramine. Each data point represents the mean ± SEM for at least four trials totaling a minimum of 40 animals.
(TIF)

Figure S2 *tyra-2* and *tyra-3* mutant animals paralyze on exogenous tyramine. (A) *tyra-2* and *tyra-3* mutants become immobilized on plates containing 30 mM exogenous tyramine similar to wild-type. (B) Two additional SER-2 rescue strains (10 ng/µl injection) also rescue the immobilization resistance phenotype of *ser-2* mutants. Rescue denotes the transgenic line *Pser-2*::SER-2*; ser-2(pk1357).* Each data point represents the mean ± SEM for at least five trials totaling a minimum of 50 animals.
(TIF)

Figure S3 *Pser-2*::GFP and *Plgc-55*::mCherry are expressed in different cells. (A–C) Transgenic adult animal co-expressing (A) *Pser-2*::GFP and (B) *Plgc-55*::mCherry. (C) Merge. Head muscle expression of *Pser-2*::GFP does not overlap with neck muscle expression of *Plcg-55*::mCherry. Unlike *lgc-55* mutants, *ser-2* mutants do suppress head movements in response to touch. *ser-2* mutants occasionally reinitiate head movements before the reinitiation of forward locomotion (unpublished observation), which may suggest a role for *ser-2* in head muscles.
(TIF)

Figure S4 SER-2 acts in a Gα_o_ pathway in GABAergic neurons. (A) Shown is the percentage of animals that display sustained locomotion on 30 mM exogenous tyramine (see also Figure 2E). *unc-25* (GABA deficient) mutants and *unc-25; ser-2(pk1357)* double mutants are not resistant to the paralytic effects of exogenous tyramine. Expression of SER-2 in all GABAergic neurons (*Punc-47*::SER-2) restores sensitivity of *ser-2* mutants to exogenous tyramine. (B) Expression of GOA-1/Gα_o_ or EAT-16/RGS in all GABAergic neurons (*Punc-47*::GOA-1 or *Punc-47*::EAT-16) partially restores sensitivity to exogenous tyramine in the respective *goa-1* and *eat-16* mutants. Each data point represents the mean percentage of animals immobilized by tyramine each minute for 20 min ± SEM for at least three trials, totaling a minimum of 30 animals.
(TIF)

Figure S5 Ablation of GABAergic DD neurons impair ventral omega turns. Average number of closed omega turns made by animals with either VD (*n* = 7) or DD (*n* = 12) neurons ablated or mock ablated animals (*n = *13). Error bars represent SEM, ***p<0.001, two-tailed Student's *t* test.
(TIF)

Figure S6 Reversal length after gentle anterior touch. Distribution of the number of backward body bends in response to anterior touch of wild-type and *ser-2* mutants. *n*≥250 animals per genotype.
(TIF)

Movie S1 Laser ablation of the DD GABAerigic motor neurons results in a dorsal bias during forward locomotion. The operated animal locomotes in a circular pattern with its ventral side (location of the vulva) facing the outside of the circles.
(MOV)

Movie S2 Laser ablation of the VD GABAerigic motor neurons results in a ventral bias during forward locomotion. The operated animal locomotes in a circular pattern with its ventral side (location of the vulva) facing the inside of the circles.
(MOV)

Movie S3 Tracking of a [*Pflp-13::*ChR2; *Pflp-13*::NpHR] single worm's locomotion and spatial illumination of the ventral nerve cord using the CoLBeRT system [49]. Inhibition of the DD motor neurons with green light activation of NpHR causes a dorsal bend. Activation of the DD motor neurons with blue light activation of ChR2 causes a ventral bend.
(MOV)

Movie S4 A wild-type animal executing a complete omega turn in response to an anterior touch. Following a long reversal, the animal makes a deep ventral turn where the head of the animal touches and slides along the ventral side of the body and resumes forward locomotion in the direction opposite its original trajectory.
(MOV)

Movie S5 A *ser-2* mutant animal does not execute a complete omega turn in response to an anterior touch. Following a long reversal, the animal's head fails to touch the ventral side of the body.
(MOV)

## Abbreviations

ACh: acetylcholine
cAMP: cyclic adenosine monophosphate
ChR2: Channelrhodopsin-2
CoLBeRT: Control of Locomotion and Behavior in Real Time
DAGθ: diacylglycerol kinase theta
Gα: G protein-alpha subunit
GABA: gamma-aminobutyric acid
GPCR: G-protein coupled receptor
ICF: intracellular fluid
IPSC: inhibitory post-synaptic current
NGM: nematode growth media
NMJ: neuromuscular junction
NpHR: Halorhodopsin
RGS: regulator of G-protein signaling
t_i50_: time to immobilize 50% of the animals
wt: wild type
