# Supplementary figures and images for: Monoaminergic Orchestration of Motor Programs in a Complex C. elegans Behavior
Source: PLoS Biol. 2013 Apr 2;11(4):e1001529. doi: 10.1371/journal.pbio.1001529 (PMC3614513; doi:10.1371/journal.pbio.1001529)

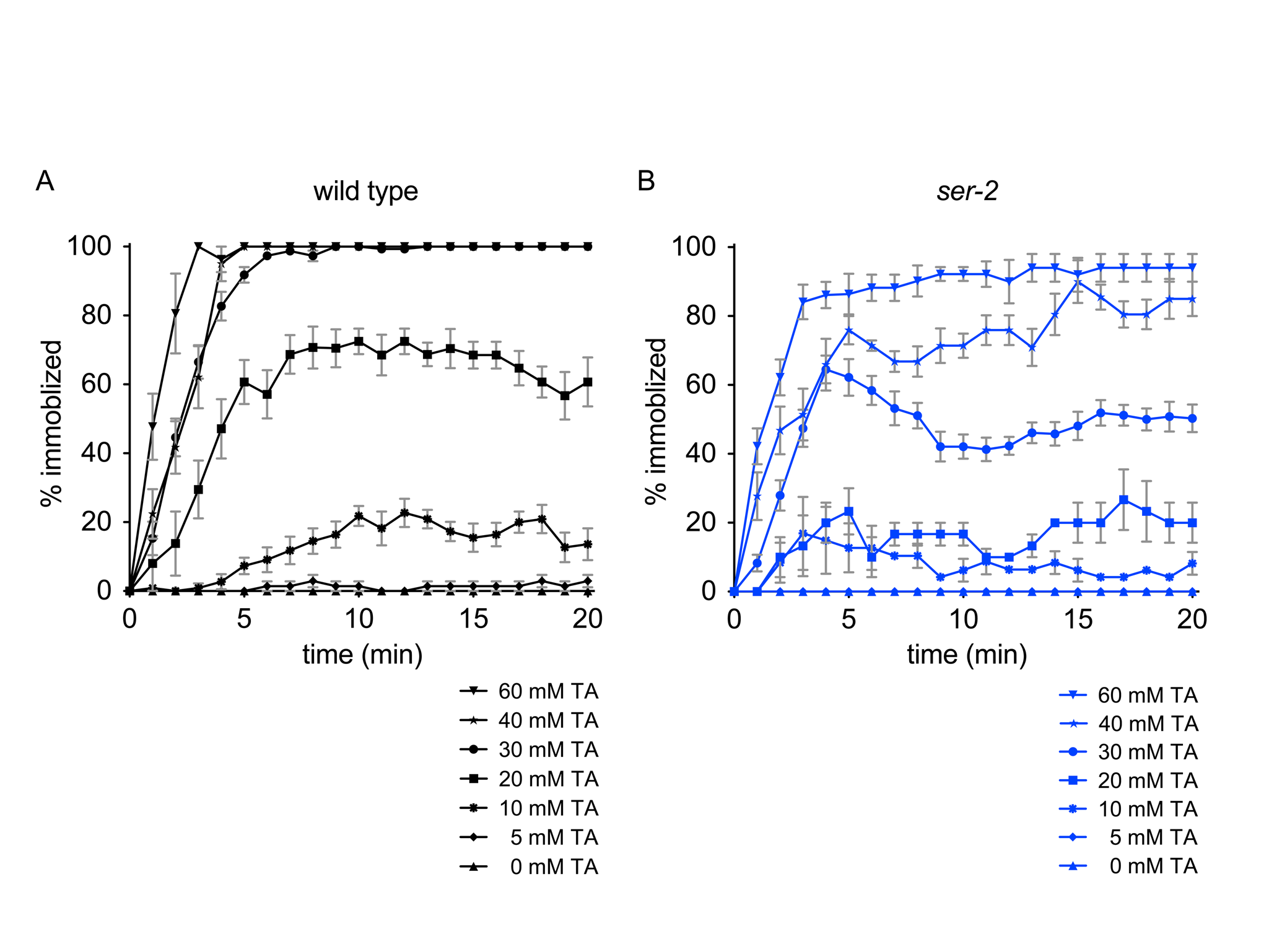

Supplement: Figure S1 — C. elegans become immobilized on exogenous tyramine in a dose-dependent manner. (A) Wild-type animals become immobilized within 5 min on 30 mM tyramine (also see Pirri et al., 2009 [22]). (B) ser-2 mutants are resistant to body immobilization compared to wild-type, but become immobilized by 60 mM tyramine. Each data point represents the mean ± SEM for at least four trials totaling a minimum of 40 animals. (TIF) [file pbio.1001529.s001.tif]

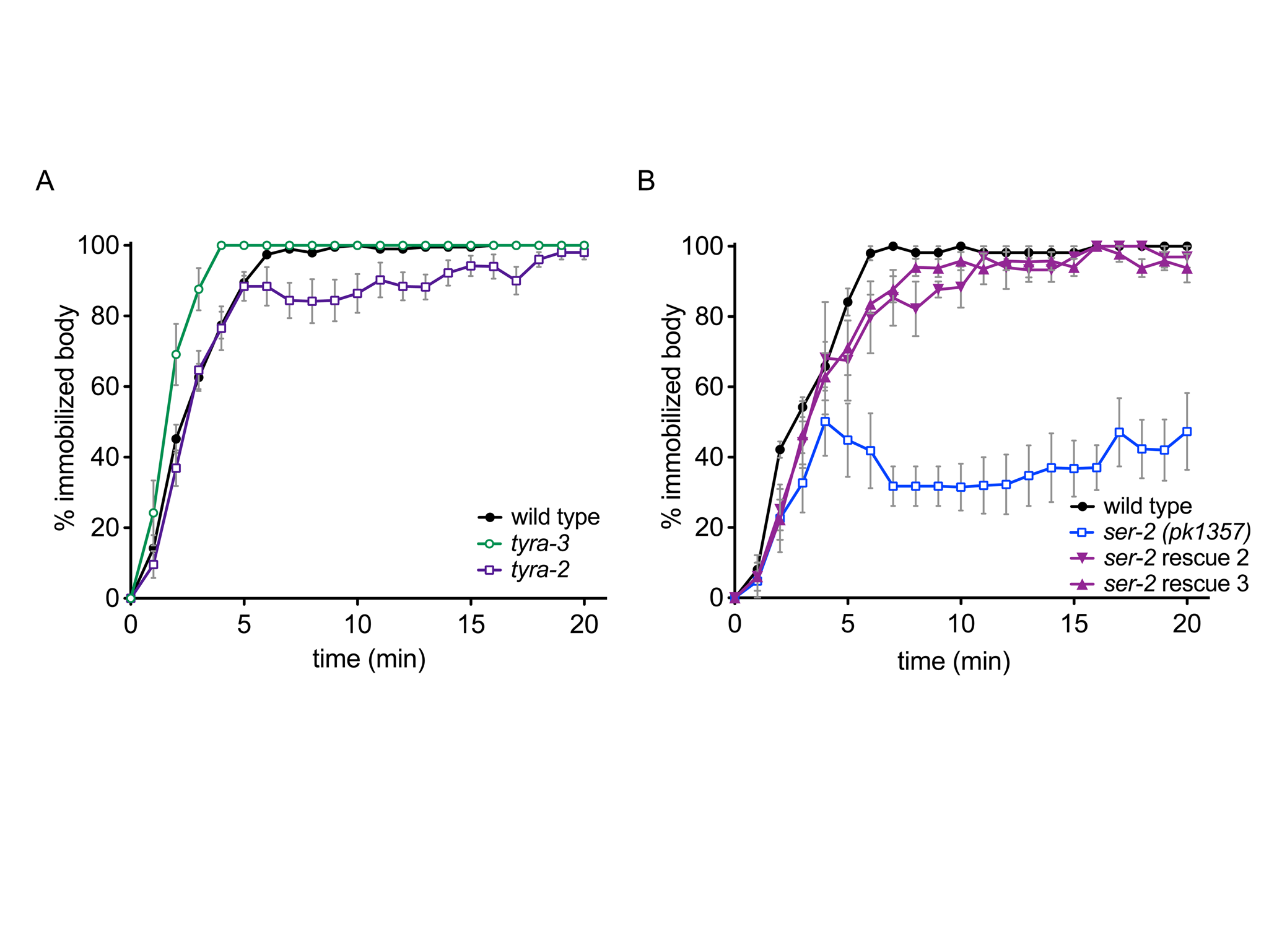

Supplement: Figure S2 — tyra-2 and tyra-3 mutant animals paralyze on exogenous tyramine. (A) tyra-2 and tyra-3 mutants become immobilized on plates containing 30 mM exogenous tyramine similar to wild-type. (B) Two additional SER-2 rescue strains (10 ng/µl injection) also rescue the immobilization resistance phenotype of ser-2 mutants. Rescue denotes the transgenic line Pser-2::SER-2; ser-2(pk1357). Each data point represents the mean ± SEM for at least five trials totaling a minimum of 50 animals. (TIF) [file pbio.1001529.s002.tif]

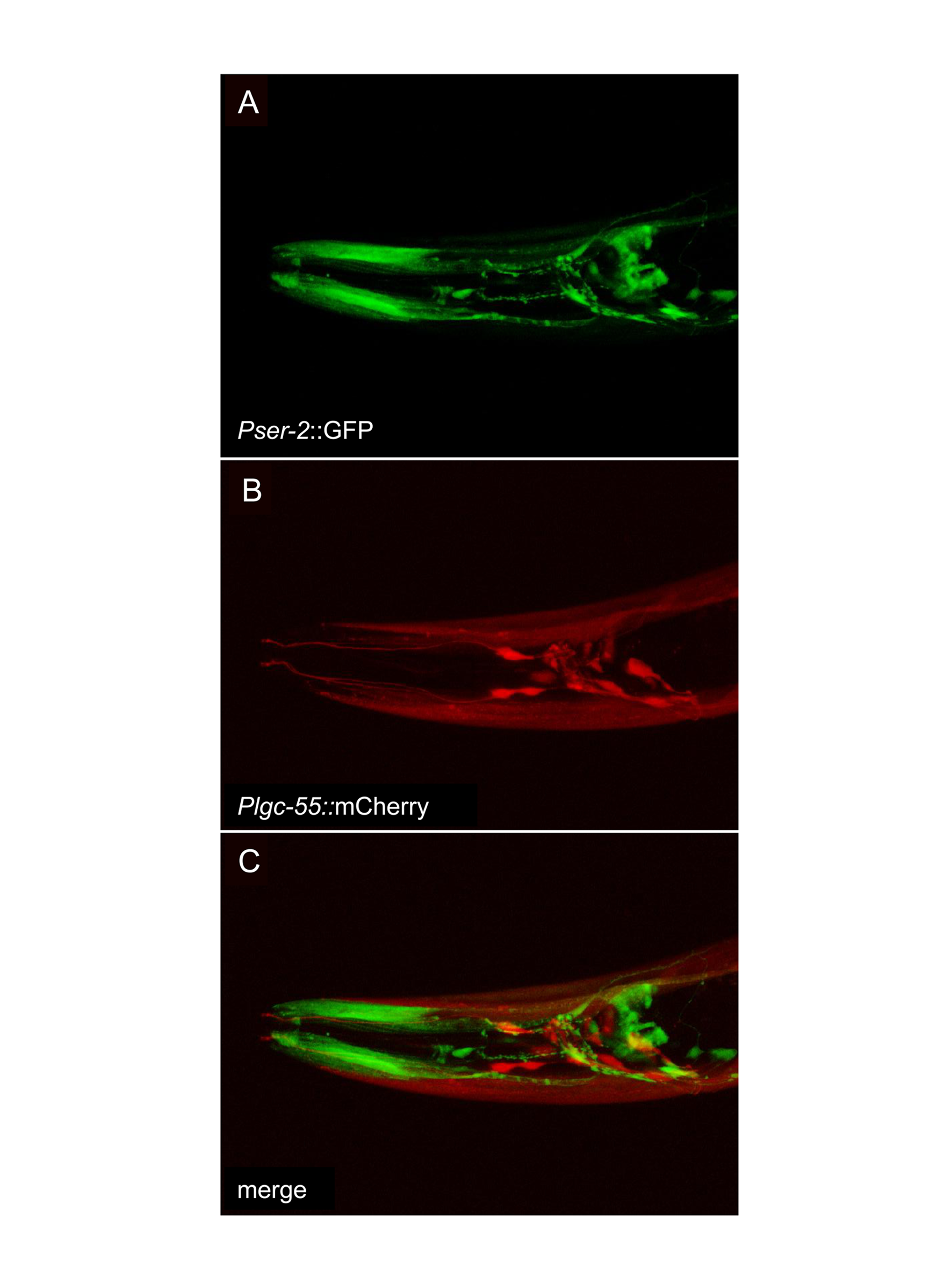

Supplement: Figure S3 — Pser-2::GFP and Plgc-55::mCherry are expressed in different cells. (A–C) Transgenic adult animal co-expressing (A) Pser-2::GFP and (B) Plgc-55::mCherry. (C) Merge. Head muscle expression of Pser-2::GFP does not overlap with neck muscle expression of Plcg-55::mCherry. Unlike lgc-55 mutants, ser-2 mutants do suppress head movements in response to touch. ser-2 mutants occasionally reinitiate head movements before the reinitiation of forward locomotion (unpublished observation), which may suggest a role for ser-2 in head muscles. (TIF) [file pbio.1001529.s003.tif]

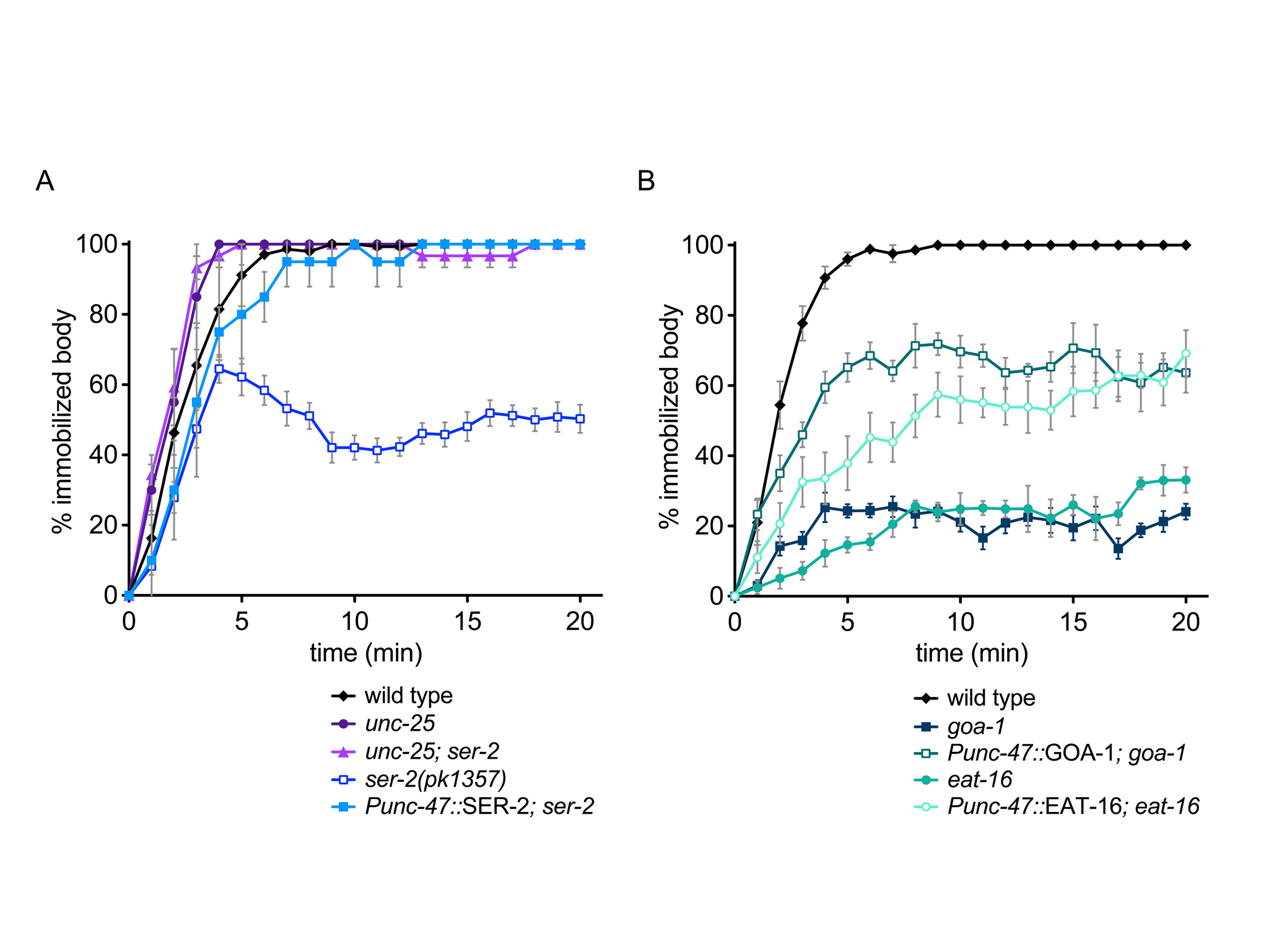

Supplement: Figure S4 — SER-2 acts in a Gαo pathway in GABAergic neurons. (A) Shown is the percentage of animals that display sustained locomotion on 30 mM exogenous tyramine (see also Figure 2E). unc-25 (GABA deficient) mutants and unc-25; ser-2(pk1357) double mutants are not resistant to the paralytic effects of exogenous tyramine. Expression of SER-2 in all GABAergic neurons (Punc-47::SER-2) restores sensitivity of ser-2 mutants to exogenous tyramine. (B) Expression of GOA-1/Gαo or EAT-16/RGS in all GABAergic neurons (Punc-47::GOA-1 or Punc-47::EAT-16) partially restores sensitivity to exogenous tyramine in the respective goa-1 and eat-16 mutants. Each data point represents the mean percentage of animals immobilized by tyramine each minute for 20 min ± SEM for at least three trials, totaling a minimum of 30 animals. (TIF) [file pbio.1001529.s004.tif]

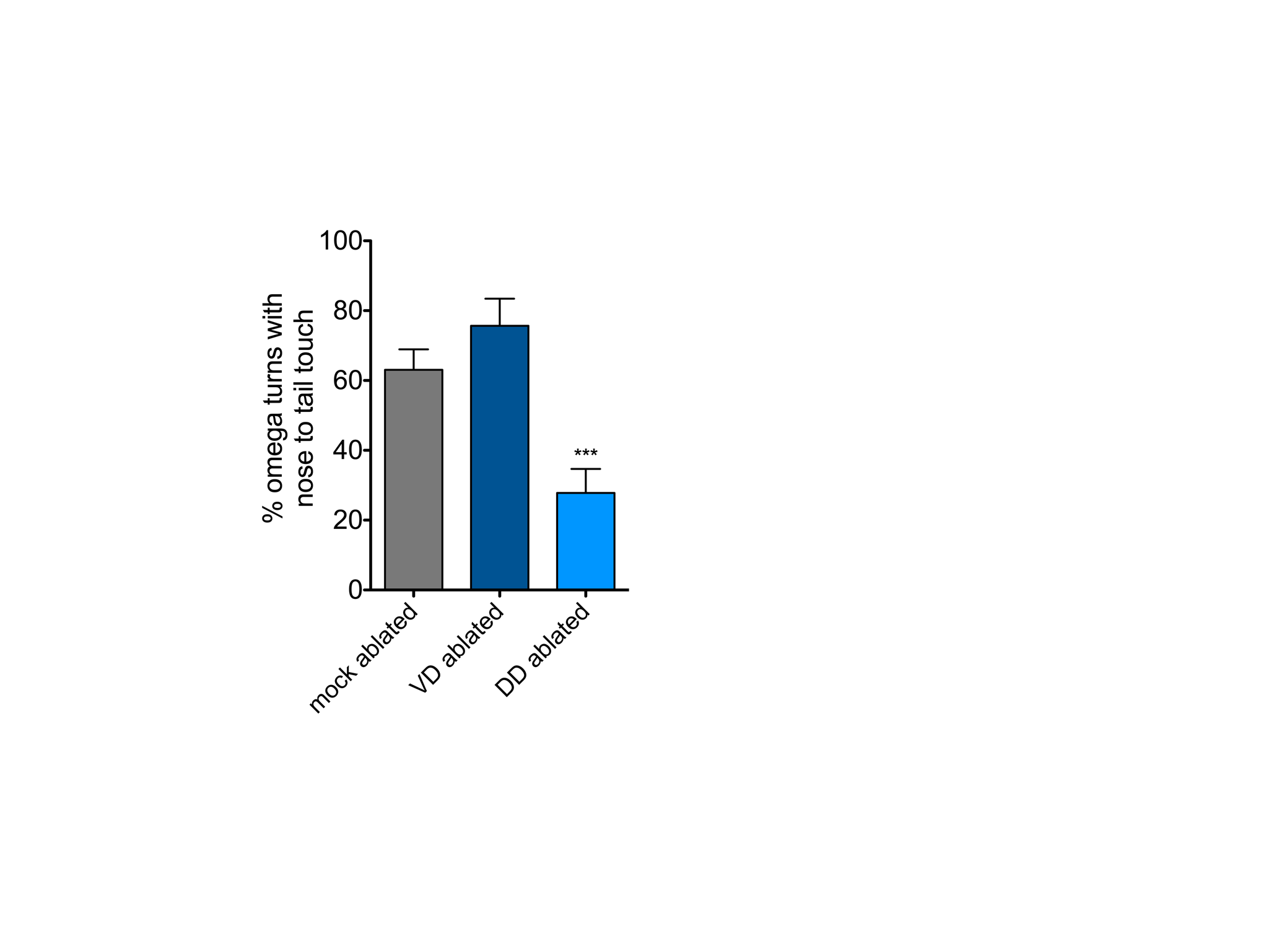

Supplement: Figure S5 — Ablation of GABAergic DD neurons impair ventral omega turns. Average number of closed omega turns made by animals with either VD (n = 7) or DD (n = 12) neurons ablated or mock ablated animals (n = 13). Error bars represent SEM, ***p<0.001, two-tailed Student's t test. (TIF) [file pbio.1001529.s005.tif]

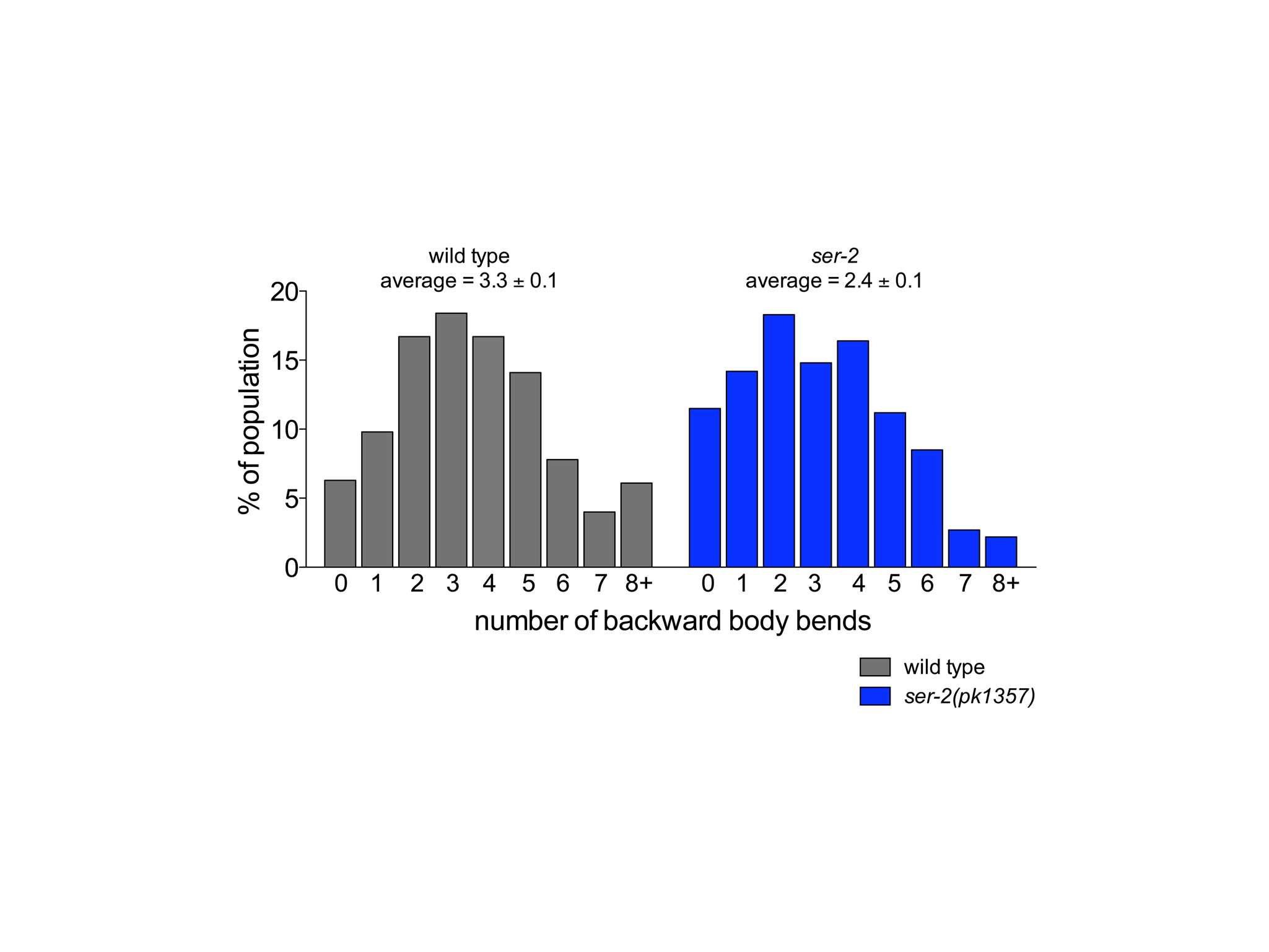

Supplement: Figure S6 — Reversal length after gentle anterior touch. Distribution of the number of backward body bends in response to anterior touch of wild-type and ser-2 mutants. n≥250 animals per genotype. (TIF) [file pbio.1001529.s006.tif]
